# Angiography- and IVUS-tip-detection-guided 3D wiring for CTO and non-CTO percutaneous coronary interventions

**DOI:** 10.1007/s12928-026-01282-2

**Published:** 2026-04-28

**Authors:** Atsunori Okamura, Ryohei Yoshikawa, Kota Tanaka, Satoshi Suzuki, Masato Ishikawa, Hiroyuki Nagai, Tomohiro Yamasaki, Kazunori Yamaji, Katsutoshi Kawamura, Naotaka Okamoto, Takayuki Ishihara, Wataru Nagamatsu, Takeshi Serikawa, Yoshiaki Ito, Heitaro Watanabe

**Affiliations:** 1 Cardiovascular Center, Sakurabashi Watanabe Advanced Healthcare Hospital, NAKANOSHIMA QROSS 4-3-51 Nakanoshima, Kita-ku, 530-0005 Osaka, Japan; 2 Department of Cardiovascular Medicine, Sanda City Hospital, Sanda, Japan; 3 Department of Cardiology, Kindai University Nara Hospital, Nara, Japan; 4 Department of Cardiology, Fujita Health University, Aichi, Japan; 5 Division of Cardiovascular Medicine, Department of Internal Medicine, Kurume University School of Medicine, Kurume, Japan; 6 Department of Radiology, Kindai University Nara Hospital, Nara, Japan; 7 Division of Cardiology, Osaka Rosai Hospital, Sakai, Osaka Japan; 8 Cardiovascular Center, Kansai Rosai Hospital, Amagasaki, Hyogo Japan; 9 Department of Cardiology, Hokusetsu General Hospital, Takatsuki, Osaka Japan; 10 Department of Cardiology, Fukuoka Wajiro Hospital, Fukuoka, Japan; 11 Cardiovascular Center, Saiseikai Yokohama City Eastern Hospital, Yokohama, Kanagawa Japan

**Keywords:** Chronic total occlusion, 3D wiring, Tip detection method

## Abstract

Precise navigation of a guidewire tip to a target within the three-dimensional (3D) structure of a coronary artery—controlled from 140 cm away—requires “3D wiring,” a technique involving wire manipulation based on 3D imaging. Hypothesizing that 3D wiring enables the standardization of wiring techniques for chronic total occlusion (CTO) intervention, we have established a 3D wiring method utilizing fluoroscopy and intravascular ultrasound (IVUS). As a first step to visualize guidewire movement within CTO lesions, we collaborated with Terumo Corporation to develop the Navifocus WR in 2012, a CTO-specific IVUS featuring a small diameter and a short tip. Observing guidewire movement during CTO PCI using this IVUS provided the insights necessary to understand how the shaft and tip must be visualized to facilitate effective 3D wiring. We first established angiography-guided 3D wiring using “3D imaging rules” in 2014. Subsequently, to achieve real-time IVUS-guided 3D wiring, we devised the tip detection (TD) method in 2017 and developed the AnteOwl WR IVUS (Terumo Corporation)—a short-tip, pullback-compatible catheter—in 2019, establishing a strategic approach for TD-intra-plaque tracking. Contrary to the traditional view that IVUS guidance is limited to intra-plaque tracking, our 2021 findings show that re-entry is routinely feasible through TD-guided perpendicular puncture (TD-ADR: Antegrade Dissection and Re-entry). This evolution elevated the TD method into a more robust technique for wire crossing. This review aims to systematically organize the theory of 3D wiring and disseminate this knowledge to the global community.

**Supplementary Information:**

The online version contains supplementary material available at https://doi.org/10.1007/s12928-026-01282-2.

## Introduction

In percutaneous coronary intervention (PCI), guidewire manipulation has been performed only via two-dimensional (2D) manipulation in most situations. For guidewire manipulation in non-chronic total occlusion (CTO) lesions, 2D guidewire manipulation is sufficient in most situations. However, for guidewire manipulation in CTO lesions, it is necessary to advance the guidewire accurately to the target in a three-dimensional (3D) structure, and 2D guidewire manipulation gives rise to uncertainties. To overcome this problem, the parallel wire technique and the retrograde approach (i.e., retrograde wire escalation [RWE]) were developed as an extension of 2D guidewire manipulation. In particular, RWE, by using the reverse controlled antegrade and retrograde tracking (CART) technique [1, 2], eliminated the need to accurately advance the wire to the target in a 3D structure and greatly contributed to lesion crossing with 2D guidewire manipulation.

**Fig. 1:**
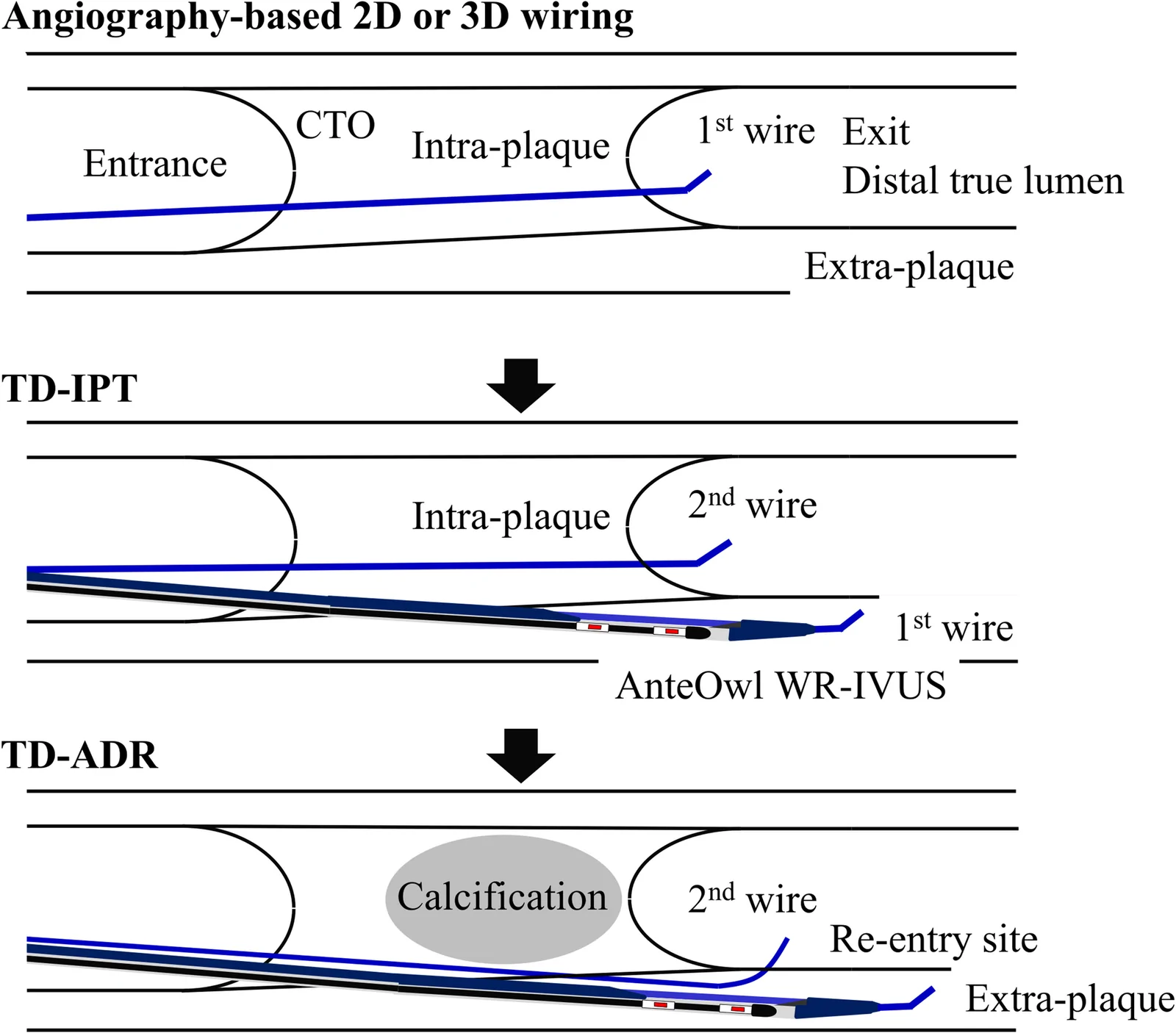
Evolution of 3D wiring. *2D* two-dimensional, *3D* three-dimensional, *CTO* chronic total occlusion, *IVUS* intravascular ultrasound, *TD-IPT* tip detection-intraplaque tracking, *TD-ADR* tip detection-antegrade dissection re-entry

About 15 years ago, we speculated that establishing 3D wiring—that is, manipulating the guidewire with 3D movements against a target with a 3D structure—could solve the essence of the problem and allow for the standardization of the guidewire manipulation method for CTO PCI. First, to make it easier to observe the movement of the guidewire inside the CTO lesions, we developed the CTO-specific intravascular ultrasound (IVUS), Navifocus WR (small-diameter, short-tip; Terumo Corp., Tokyo, Japan) in 2010 [3]. Through CTO PCI procedures using this IVUS, we became convinced of the necessity of the “3D wiring method” to guide the guidewire tip to the target based on a 3D image. At that time, there was no IVUS that combined a short tip with a pullback system; therefore, we established angiography-guided 3D wiring in 2014 [4, 5]. Then, we devised the tip detection (TD) method in 2017 and developed the AnteOwl WR IVUS (Terumo Corp.), which combines a short tip with a pullback system, in 2019 to standardize intra-plaque tracking using the TD method [6]. Our initial goal was to establish this TD-intra-plaque tracking (TD-IPT). With increasing experience in the TD-IPT procedure, we incidentally discovered TD-antegrade dissection and re-entry (TD-ADR) in 2021. This technique enables re-entry from the extra-plaque space to the intra-plaque area via a perpendicular puncture at the optimal point using the TD method (Fig. 1) [7, 8]. The integration of TD-ADR—a technique achieving high-precision re-entry—with angiography-guided 3D wiring and IVUS-guided TD-IPT, which together represent the pinnacle of accurate intra-plaque wiring, has established a highly precise and robust foundation for the antegrade approach in CTO PCI.

## Positioning of the 3D wiring method in CTO PCI strategy

**Fig. 2:**
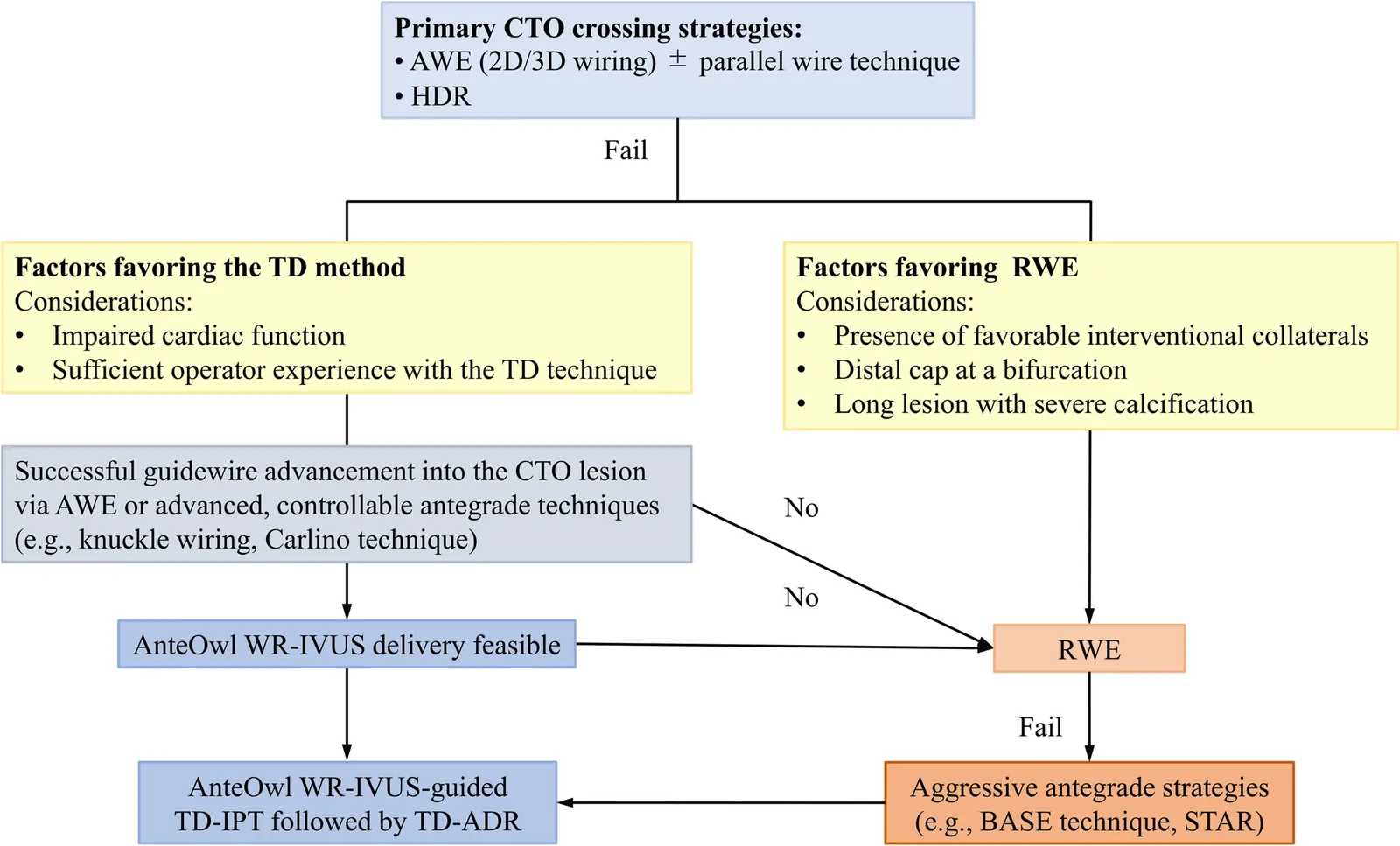
Hybrid tip detection-retrograde wire escalation algorithm. *AW*E antegrade wire escalation, *BASE* balloon-assisted subintimal entry, *CTO* chronic total occlusion, *IVUS* intravascular ultrasound, *HDR* hydraulic dissection re-entry, *TD* tip detection, *TD-ADR* tip detection-antegrade dissection re-entry, *TD-IPT* tip detection-intraplaque tracking, *RWE* retrograde wire escalation

In the latest CTO PCI strategy from the AP-CTO club, if guidewire passage is difficult with antegrade wire escalation (AWE), the options of parallel wiring, Stingray-ADR (Boston Scientific Corp., Natick, Massachusetts) [9], or IVUS-guided wiring with the TD method using a CTO-specific IVUS are considered equivalent [10]. We have been performing AnteOwl WR IVUS-guided wiring using the TD method since 2019. Based on this experience, Fig. 2 presents a strategy that incorporates TD-based IVUS-guided wiring. This strategy references the Western Hybrid Algorithm [11] and the AP-CTO Club Global CTO Algorithm, while also taking into account the recently emerging Hydrodynamic Contrast Recanalization (HDR) [12] and the combined HDR-TD method reported by Kaneko and Fujita [13].

In Japan, as TD-ADR becomes more widespread, Stingray-ADR is now rarely performed from the standpoint of procedural accuracy [14]. Therefore, if AWE (via 2D/3D wiring) or HDR fails to cross the lesion, it becomes a binary choice between RWE and TD-IVUS guided wiring. Factors favoring an RWE approach include the presence of a good interventional retrograde channel, a distal exit at a bifurcation, and a long lesion with severe calcification. Conversely, factors favoring the TD method include poor cardiac function and proficiency in this method. At this time, the operator’s skill is a significant factor, especially for the TD method. It is crucial to acquire the skills to perform not only TD-ADR but also TD-IPT. If the decision is made to proceed with the TD method based on the aforementioned factors, the antegrade guidewire should be advanced further distally as needed to facilitate IVUS observation of the CTO lesion. It is crucial to consider the use of a polymer-jacketed wire, such as a Gladius EX (Asahi Intecc, Aichi, Japan), to ensure the guidewire remains intravascular. If necessary, the strategy should transition to advanced, controllable antegrade techniques such as knuckle wiring or Carlino [15]. Once the guidewire can be advanced into the CTO body, the AnteOwl WR IVUS is inserted to move on to the TD method.

Similarly, RWE is performed if it is selected based on the aforementioned factors, or if the TD method was initially chosen but the IVUS could not be advanced antegradely into the CTO body. If RWE is difficult, more aggressive antegrade techniques, such as balloon-assisted subintimal entry (BASE) [16] or the subintimal tracking and re-entry (STAR) technique [15], are considered, and then the AnteOwl WR IVUS is inserted into the CTO lesion to perform the TD method.

## Fundamentals of the 3D wiring technique for CTO lesions

**Fig. 3:**
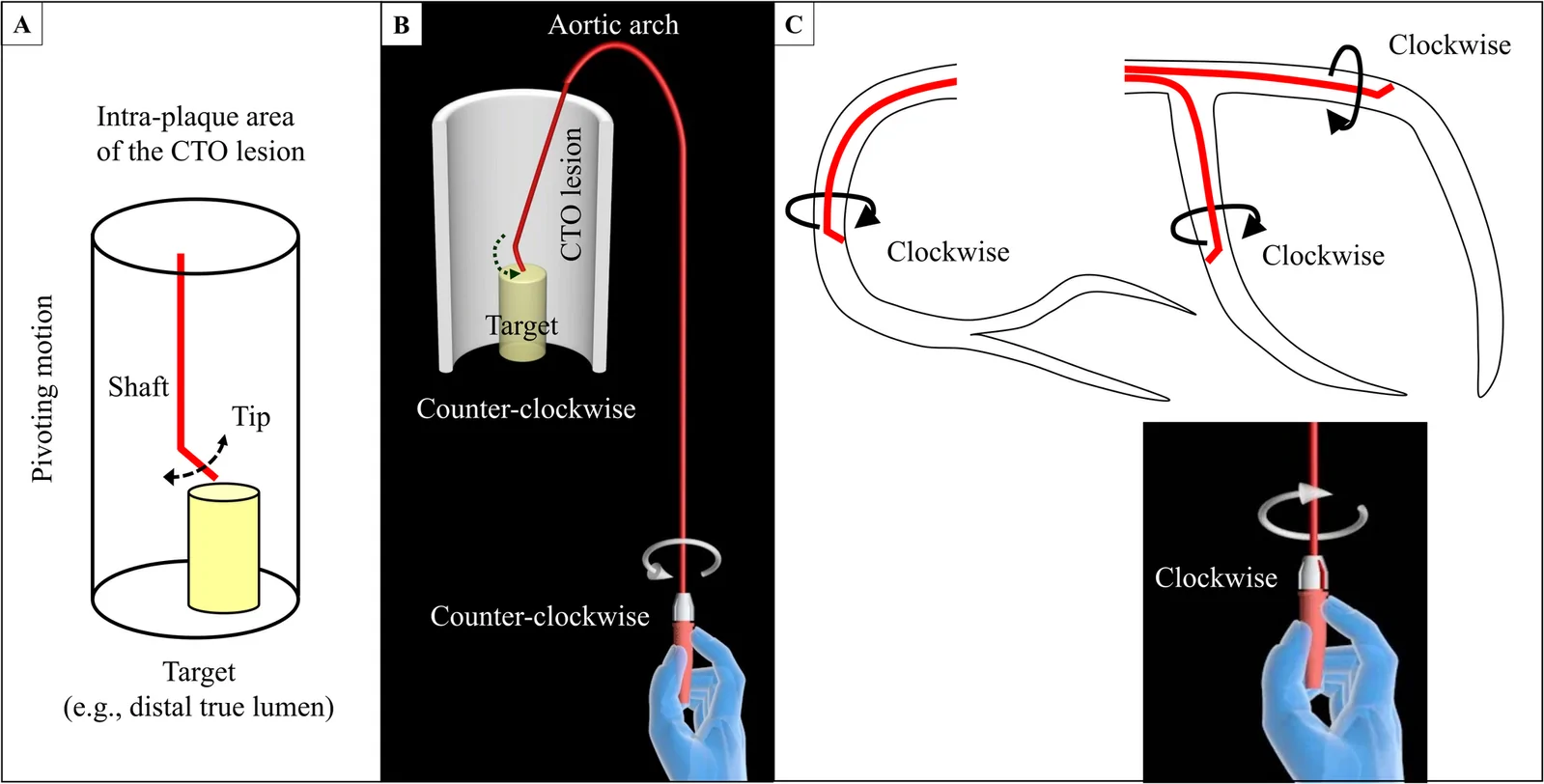
Core principles and rotational dynamics of the 3-dimentional wiring technique in chronic total occlusion intervention. (**A**) The wire shaft is fixed, and the wire tip rotates around the shaft within the intraplaque space of the CTO lesion. (**B**) Precise control of the wire tip from a torquer located 140 cm away is essential. Although the torquer and the tip rotate in the same direction, they appear visually reversed because the guidewire reverses its orientation at the aortic arch. (**C**) The operator must be able to mentally synchronize the rotation of the torquer with the movement of the tip across all coronary segments. *CTO* chronic total occlusion

First, we will describe how a CTO stiff guidewire moves inside a CTO lesion (Fig. 3A). Usually, the guidewire is shaped with only a first curve of 1 mm at a 45-degree angle at its tip. When this guidewire is rotated inside the CTO tissue, only the tip rotates with a pivot-like motion using the shaft as an axis. The 3D wiring technique utilizes this pivot-like movement. However, as the tissue breaks down with guidewire manipulation, it becomes difficult for the shaft to remain fixed as an axis by the tissue. Consequently, the shaft also begins to move, which reduces the precision of guidewire manipulation in the 3D wiring technique.

To perform the 3D wiring technique, it is essential to understand its specific guidewire manipulations. Advancing a guidewire inside the CTO lesion involves only two actions: rotating and pushing. While 2D wiring involves advancing a low-gram (approx. 3 g) CTO stiff guidewire by simultaneously rotating and pushing moderately, the 3D wiring technique requires the operator to clearly distinguish between these two actions to advance the guidewire toward the target. In other words, this technique requires the skill to control the guidewire tip from a torque device 140 cm away, based on a 3D image, to advance it toward the target (Fig. 3B).

Whenever performing 3D wiring, 1:1 (one-to-one) rotational synchronization between the torque device and the guidewire tip is essential. When you turn the torque device clockwise, you must be able to visualize the tip also turning clockwise inside the coronary artery. This direction of rotation is defined as viewed from behind the torque device. However, since the guidewire reverses its orientation at the aortic arch, the visual direction of rotation becomes inverted, which often presents the first hurdle for operators (Fig. 3B). For example, in the mid-segment of the right coronary artery, which runs vertically, if the guidewire tip is directed to the right, the torquer must be rotated clockwise to direct the tip towards you. In the proximal segment of the left anterior descending artery, which runs horizontally, if the guidewire tip is directed superiorly, the torquer must be rotated clockwise to direct the tip towards you (Fig. 3C). By performing this manipulation even in PCI for non-CTO, open vessels, an operator can naturally train their mind to synchronize the rotation of the torque device with the movement of the guidewire tip. Furthermore, as will be mentioned later, practicing this technique will improve and stabilize guidewire manipulations in open vessels.

Next, we will discuss guidewire and guiding catheter selection for performing the 3D wiring technique. The fundamental principle of 3D wiring is to advance the guidewire by pointing its tip in the desired direction and then pushing. This maneuver requires a guidewire with two key characteristics: (1) the ability to advance with appropriate deflection upon pushing, and (2) excellent torque response. Based on the author’s experience, the guidewire selection is as follows: (1) Angiography-based 3D wiring: The first-choice guidewires are the Conquest Pro 9 g (shaped with a 0.8 mm, 45-degree curve), the Conquest Pro 12 g, or the Conquest Pro 20 g (with a pre-shaped 1 mm, 45-degree curve, Asahi Intecc, Aichi, Japan). The Conquest Pro 9 g is suitable for manipulations in small vessels, as a 0.8 mm curve can be shaped manually. The use of a Corsair microcatheter (Asahi Intecc) is essential, as it provides excellent backup support for the guidewire. (2) IVUS-based 3D wiring using the TD method: The operator advances the guidewire while observing both the tip and the target, which reduces the risk of tip stiffness. Therefore, a guidewire that advances in a predictable direction should be selected. For TD-IPT, the first-choice guidewires are the Conquest Pro 20 g and the CP12ST (Conquest Pro 12 Sharpened Tip wire developed by Tsuchikane, featuring a puncturing needle tip attached to a Conquest Pro 12 g for ADR use, Asahi Intecc) [17]. Since TD-ADR is a re-entry technique, the CP12ST, which has the highest penetration force, is the sole option in Japan. However, in countries where the CP12ST is unavailable, the Conquest Pro 20 g can be used as an alternative [8]. The Corsair is the preferred microcatheter due to its strong backup support. However, if a smaller microcatheter is required to use a balloon anchor technique at a side branch with an 8 Fr guiding catheter, the Caravel (Asahi Intecc) is selected, as it offers the best backup support among smaller-profile microcatheters.

Regarding guiding catheter selection for the 3D wiring technique, a 7 Fr or 8 Fr system is recommended to accommodate both an IVUS catheter and a microcatheter for the second wire. While a 7 Fr catheter is feasible, an 8 Fr guiding catheter is preferable considering the potential need for a balloon anchor. Generally, for CTO PCI, most operators—including the author—utilize dual access, keeping contralateral injection and a potential retrograde approach in mind. However, Tadano, Fujita, et al. have reported a single-radial-access technique based on the concept of “Minimalistic Approach with Tip detection-antegrade dissection and re-entry” [18]. This is a rational approach: in the absence of severe calcification or extreme tortuosity within the CTO lesion, it is possible to advance the guidewire inside the vessel without contralateral injection and subsequently guide the guidewire into the distal true lumen using the TD method. Therefore, this method is a viable option depending on the specific case.

## Angiography-based 3D wiring: the 3D wiring method using observation from two fluoroscopic views with the 3D imaging rule

**Fig. 4:**
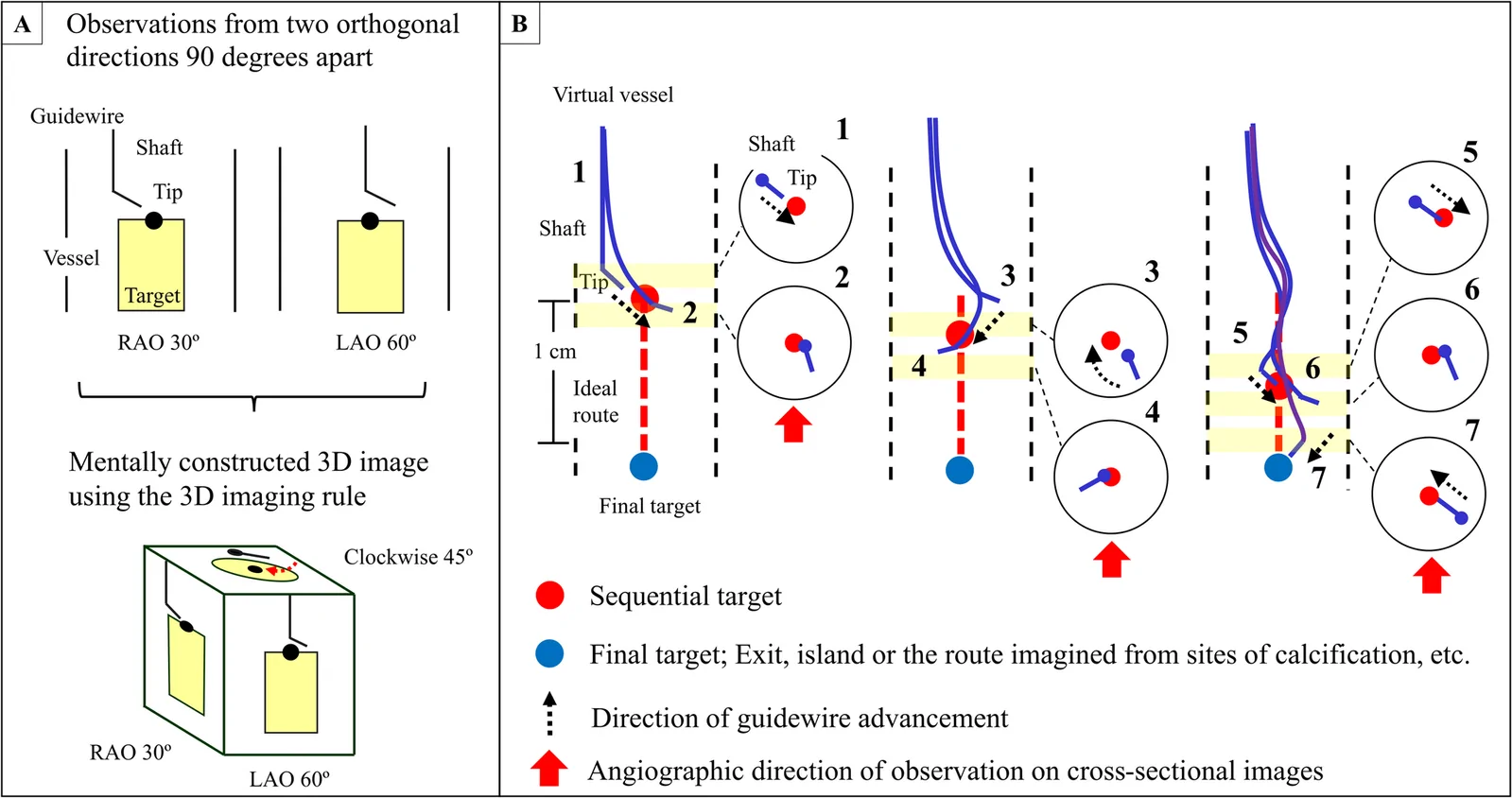
Angiography-based 3D wiring using the 3D imaging rule and the concept of sequential targets.　(**A**) By observing two fluoroscopic views and applying the 3D imaging rule, the operator can instantaneously determine the required direction of tip rotation. (**B**) Based on the concept of sequential targets, the wire tip is converged toward the final target starting from 1 cm proximally. *3D* three-dimensional

To perform angiography-based 3D wiring, it is necessary to recognize the rotational direction and angle to manipulate the guidewire in real-time from two fluoroscopic views, using the “same is front, opposite is back” 3D imaging rule (Fig. 4A) [4, 5]. By constructing this 3D image several times with the concept of sequential targets, the tip of the guidewire is converged on the target from 1 cm away (Fig. 4B).

**Fig. 5:**
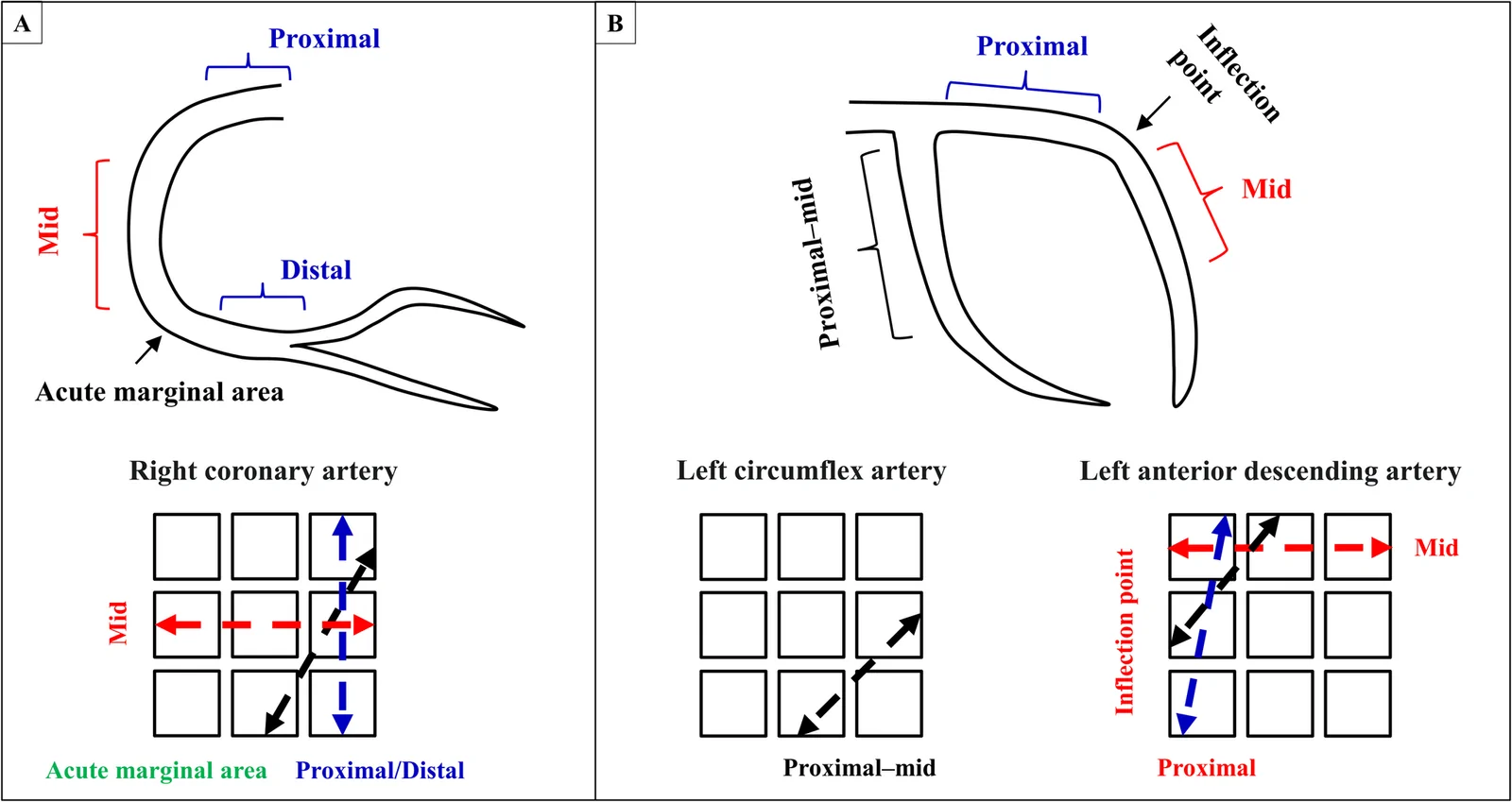
Recommended C-Arm rotations for orthogonal views of each coronary artery segment. Grid Layout: The 3 × 3 grid maps C-arm movement (Horizontal: 45° RAO to 45° LAO; Vertical: 45° cranial to 45° caudal). Optimal Projections: Recommended C-arm shift directions to achieve orthogonal viewing for each segment of the right coronary artery (**A**) and the left coronary artery (**B**). Label colors correspond to the respective arrow colors

To perform accurate guidewire manipulation on the fluoroscopic monitor, it is crucial to observe from two ideal orthogonal directions, separated by 90 degrees as much as possible. Figure 5 shows the direction (the perpendicular angle) in which the fluoroscopy detector should be rotated for orthogonal observation of each coronary artery segment, as analyzed with Philips’ computed tomography TrueView (created by Radiological Technologist, Keisuke Nishizawa). With the exception of localized coronary curves, the orthogonal directions for each segment are universal with few individual differences.

To master angiography-based 3D wiring, the most suitable method is training with either Asahi Intecc’s Rotation ETOSS [19] or Terumo’s Cubic Vessel Model. We believe that observing the guidewire manipulation in the 2D and 3D wiring videos (ESM Videos 1 and 2) [19] will clearly demonstrate the distinctions between these two approaches and aid in technical comprehension.

## Angiography-based 3D wiring: technique and clinical outcomes

In the antegrade approach, the 3D wiring technique is indicated when using a moderate stiff or stiff CTO guidewire. Suitable targets for this technique include islands inside the CTO body or the distal exit of the CTO, as well as routes that can be imaged from coronary calcification or stent struts in cases of in-stent occlusion. To construct a 3D image, the distance from the guidewire tip to the target will be within 1 cm. In the retrograde approach, 3D wiring is indicated for antegrade wiring that targets the tip of the retrograde guidewire. However, performing 3D wiring with the retrograde guidewire is difficult, as its torque response is often compromised over the long retrograde route. Nonetheless, the retrograde guidewire can be effectively guided via the IVUS-guided TD method discussed below.

The 3D wiring technique is fundamentally a pure penetrating technique. Therefore, maintaining strong backup support for the guidewire is essential. A guiding catheter of 7 Fr or 8 Fr is recommended, and a side-branch anchoring technique should be performed as needed. A microcatheter with robust support, such as the Corsair, is also preferable. For guidewire selection, if an XT-R wire (Asahi Intecc) fails to advance and the distance to the target is within 1 cm, it is appropriate to step up to a Conquest Pro 12 g or Conquest Pro 20 g. Furthermore, in situations with a well-defined route and hard tissue, such as in-stent occlusions, the use of a CP12ST should also be considered. The reason for selecting a high tip load wire from the beginning is as follows. If a guidewire with insufficient tip load is chosen, it will not advance with simple pushing. This often forces the operator to resort to a 2D wiring technique (advancing while rotating), which can create a fragile pathway or subintimal spaces inside the CTO body. If the operator then switches to a stiff Conquest wire, the compromised tissue support will prevent effective guidewire deflection, making precise manipulation difficult. Regarding guidewire tip shaping, a small, 1 mm curve with a 45-degree angle is ideal. The use of a pre-shaped Conquest wire with these specifications (1 mm, 45 degrees) is recommended. It should be noted, however, as a precaution that if the vessel course is ambiguous or if the operator is uncertain about angiographic 3D wiring, it is advisable to maintain the intravascular route using an intermediate wire, such as the Gladius EX. This approach preserves the track within the vessel and facilitates a smooth transition to the IVUS-guided TD method.

To evaluate the efficacy of angiography-based 3D wiring in CTO PCI, a single-center, retrospective study was conducted at Sakurabashi Watanabe Hospital [5]. The angiography-based 3D wiring increased the success rate of the antegrade wiring and facilitated a timely transition to the next strategy, which improved the overall procedural success rate. However, it was ineffective once the guidewire had entered the subintimal space.

## IVUS-based 3D wiring: an overview of 3D wiring for IVUS guidance

Antegrade wire navigation under IVUS guidance in CTO PCI consists of two main approaches: (1) proximal cap puncture using the TD method, visualizing the CTO entrance from a side branch, and (2) guidance of a second guidewire using the TD method under coaxial observation. The latter approach is utilized when the first guidewire has advanced into the CTO but failed to cross the lesion, allowing the IVUS catheter to be advanced over this first guidewire. While the TD method is certainly effective for proximal cap puncture of the CTO entrance in the side-branch approach, the following sections focus on the latter scenario. Herein, we describe the techniques for second guidewire guidance using coaxial IVUS (TD-IPT and TD-ADR), which represent the true essence of IVUS-guided CTO wiring.

**Fig. 6:**
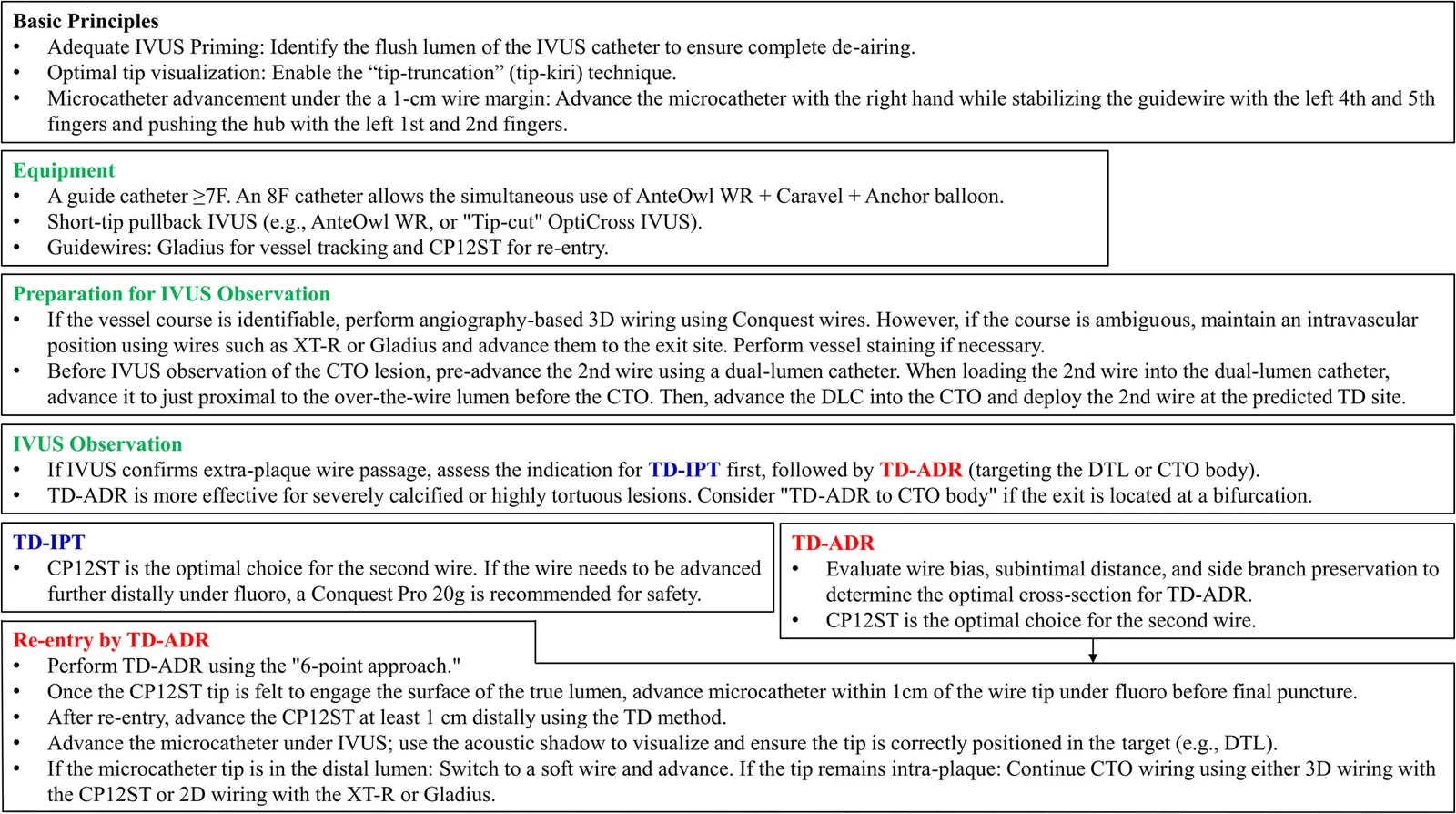
Principles of the TD method in CTO intervention. *2D* two-dimensional, *3D* three-dimensional, *CTO* chronic total occlusion, *CP12ST* Conquest Pro 12 sharpened tip, *DTL* dual-lumen catheter, *IVUS* intravascular ultrasound, *HDR* hydraulic dissection re-entry, *TD* tip detection, *TD-ADR* tip detection-antegrade dissection re-entry, *TD-IPT* tip detection-intraplaque tracking

**Fig. 7:**
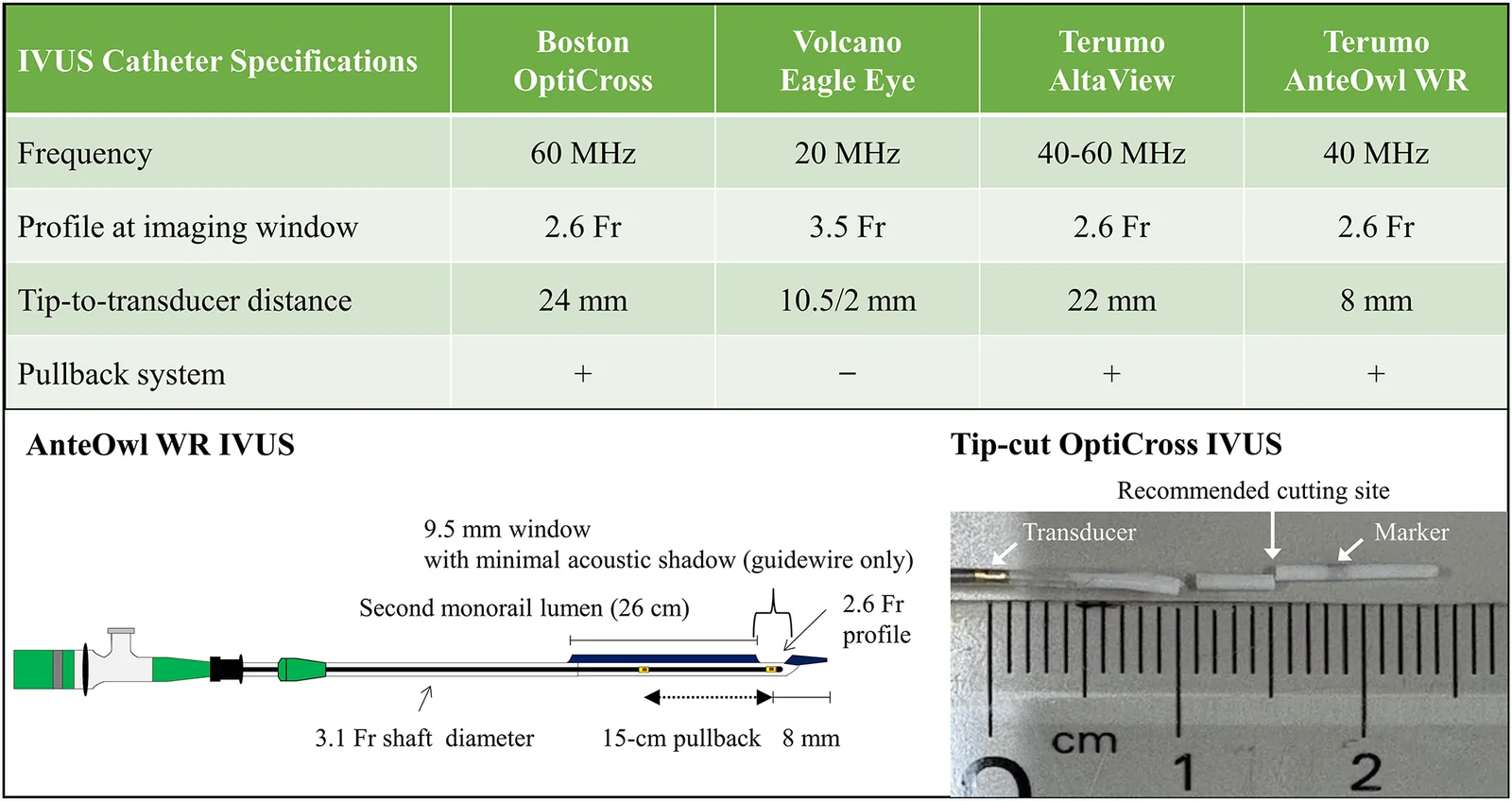
Comparison of Specifications: AnteOwl WR vs. Conventional IVUS Systems. Overview of key specifications for major IVUS systems and a detailed structural illustration of the AnteOwl WR IVUS. *IVUS* intravascular ultrasound

Figure 6 lists the key principles for the IVUS-guided TD method. These principles are described in detail in the following nine sections. In the field of IVUS-guided 3D wiring, the TD method (real-time IVUS guided wiring) is significantly more precise than the conventional IVUS-guided wiring where the guidewire’s position is confirmed after advancement [20–22]. Conventionally, detecting a guidewire with IVUS meant observing its shaft; however, by observing the tip, real-time high-precision IVUS-guided 3D wiring, that is the “TD method”, has become readily achievable [6]. The AnteOwl WR IVUS was developed to standardize the use of this TD method [6]. In addition to a 15 cm pullback system, the AnteOwl WR features a small 2.6 Fr diameter around the transducer and a 26 cm second monorail lumen to maintain pushability to allow for delivery even after bougienage with a Corsair inside a CTO lesion (Fig. 7). Currently, the AnteOwl WR is the only short-tip pullback IVUS available, and its accessibility is restricted to Japan and Hong Kong. To perform the TD method using conventional IVUS catheters, the OptiCross IVUS (Boston Scientific) is a potential candidate due to its integrated pullback system, despite a long tip-to-transducer distance exceeding 20 mm. Indeed, including cases from our institution, three successful TD-ADR procedures using the OptiCross IVUS have been reported [23–25]. However, deliverability is a major issue. Without a second monorail lumen, pushability into a CTO is limited, and the long distance from the tip to the transducer often leads to enlargement of the false lumen. In regions where the AnteOwl WR is unavailable, the only alternative is to modify an OptiCross IVUS catheter by trimming approximately 1 cm of its distal segment, including the distal marker (Fig. 7, lower right). Despite this ‘tip-cut’ modification, the catheter’s deliverability remains unexpectedly well-preserved. If delivery becomes challenging due to the shortened monorail lumen, advancing a balloon catheter over the IVUS wire to push the IVUS tip from behind can often facilitate successful tracking. Ultimately, it is hoped that short-tip pullback IVUS catheters specifically dedicated to CTO PCI will soon become globally available.

**Fig. 8:**
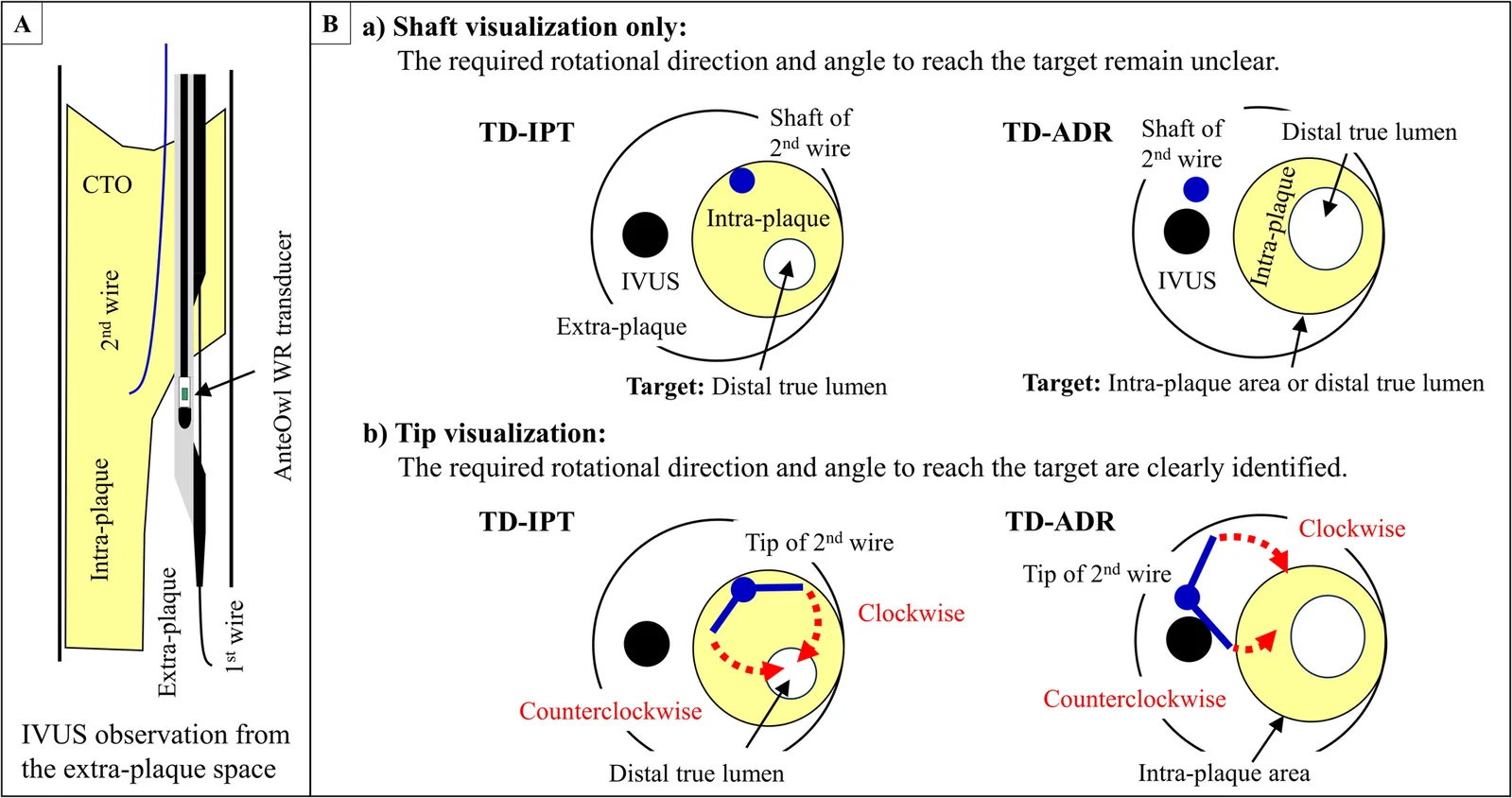
The core principle of IVUS-Guided 3D wiring: the necessity of the tip detection method. (**A**) Conceptual illustration of fluoroscopic observation. (**B**) Conceptual illustration of IVUS observation. *3D* three-dimensional, *CTO* chronic total occlusion, *IVUS* intravascular ultrasound, *TD-IPT* tip detection-intraplaque tracking, *TD-ADR* tip detection-antegrade dissection re-entry

The fundamental principle of the 3D wiring method, whether by angiography or IVUS, is to construct a 3D image relative to the target by separating the guidewire into its shaft and tip. As shown in Fig. 8A, the IVUS-guided wiring technique involves advancing an IVUS catheter over a first guidewire that has entered the extra-plaque space. Using IVUS imaging from this position in the extra-plaque space, a second guidewire is guided under direct visualization toward the target, such as an intra-plaque area or the distal true lumen. In IVUS-guided 3D wiring, it is impossible to determine the correct direction to rotate the guidewire (torquer) by observing the shaft alone (Fig. 8Ba). However, by observing the tip, the precise rotational direction and angle needed to steer the guidewire toward the target can be identified (Fig. 8Bb). Therefore, we named this method the tip detection method.

**Fig. 9:**
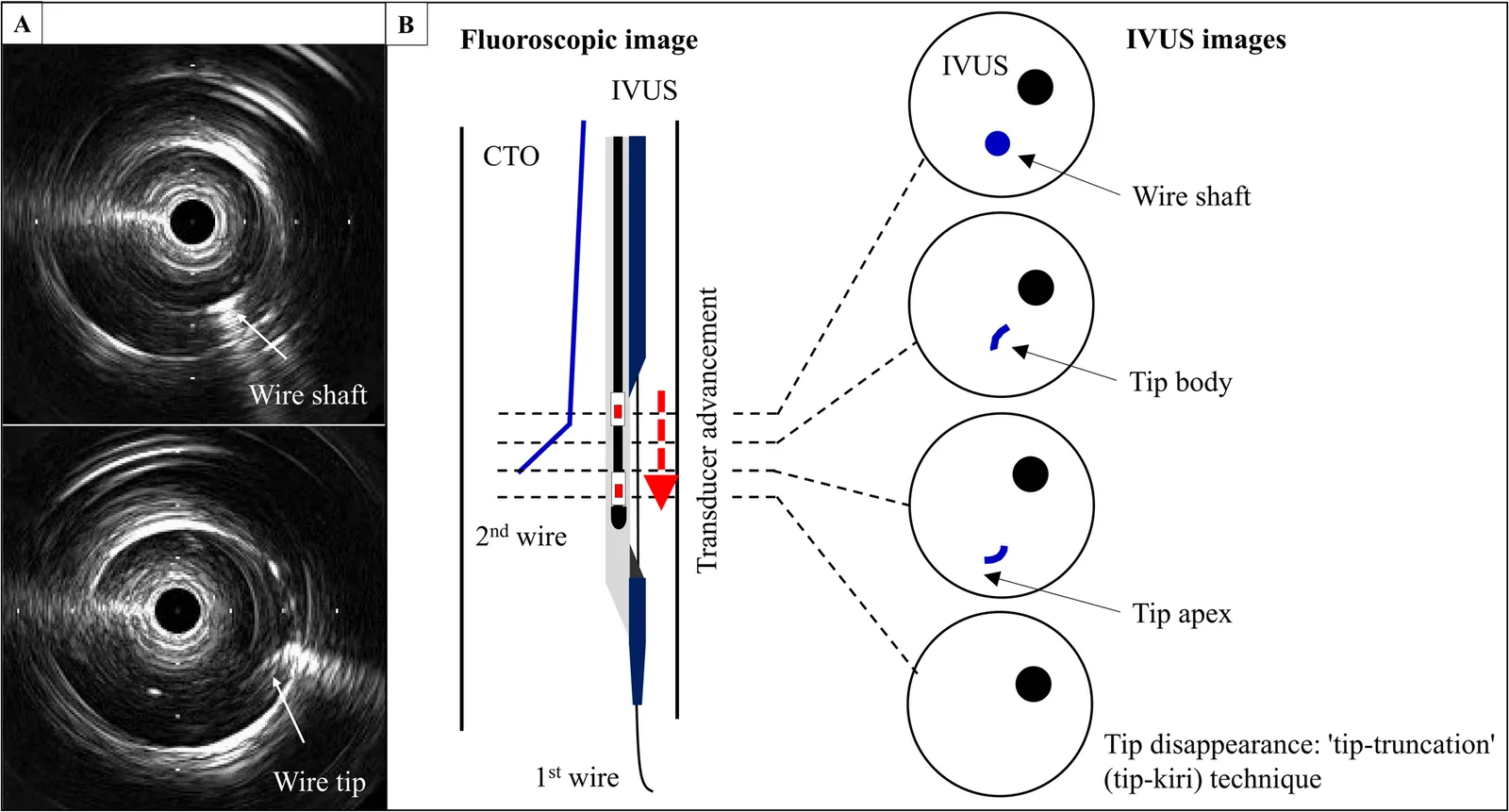
Essential tip-visualization technique for the tip detection method.　(**A**) IVUS visualization of the wire shaft and tip within an experimental model. (**B**) Illustration of the “tip-truncation” technique (tip-kiri), demonstrating the advancement of the transducer until the wire tip disappears from the IVUS image. *IVUS* intravascular ultrasound. *CTO* chronic total occlusion, *IVUS* intravascular ultrasound

As shown in Fig. 9A, when the transducer is advanced from a proximal to a distal position, the point of flexion between the shaft and the tip can be identified. The tip itself is visualized as a faint, thin image that moves along a vector corresponding to its trajectory. At this moment, as the name of the TD method implies, it is crucial to ensure that the apex of the tip is being observed. To achieve this, the wire tip should be visualized starting from its base until it disappears from the imaging plane, a maneuver referred to as the ‘tip-truncation’ (tip-kiri) technique (Fig. 9B). Furthermore, during the actual wiring procedure, this tip visualization must be performed repeatedly and in real-time, tracking the guidewire’s movement. Therefore, the second operator must master this method of tip visualization.

Constructing a 3D image for 3D wiring requires the visualization of three structures: the guidewire shaft, the guidewire tip, and the target. With angiography, the shaft and tip can be reliably visualized, but visualization of the target is often variable. In contrast, an IVUS system with a pullback function allows for the visualization of all three components, which enables easier and more precise 3D wiring.

## IVUS-based 3D wiring: procedure leading up to IVUS-based 3D wiring with the AnteOwl WR using the TD method

**Fig. 10:**
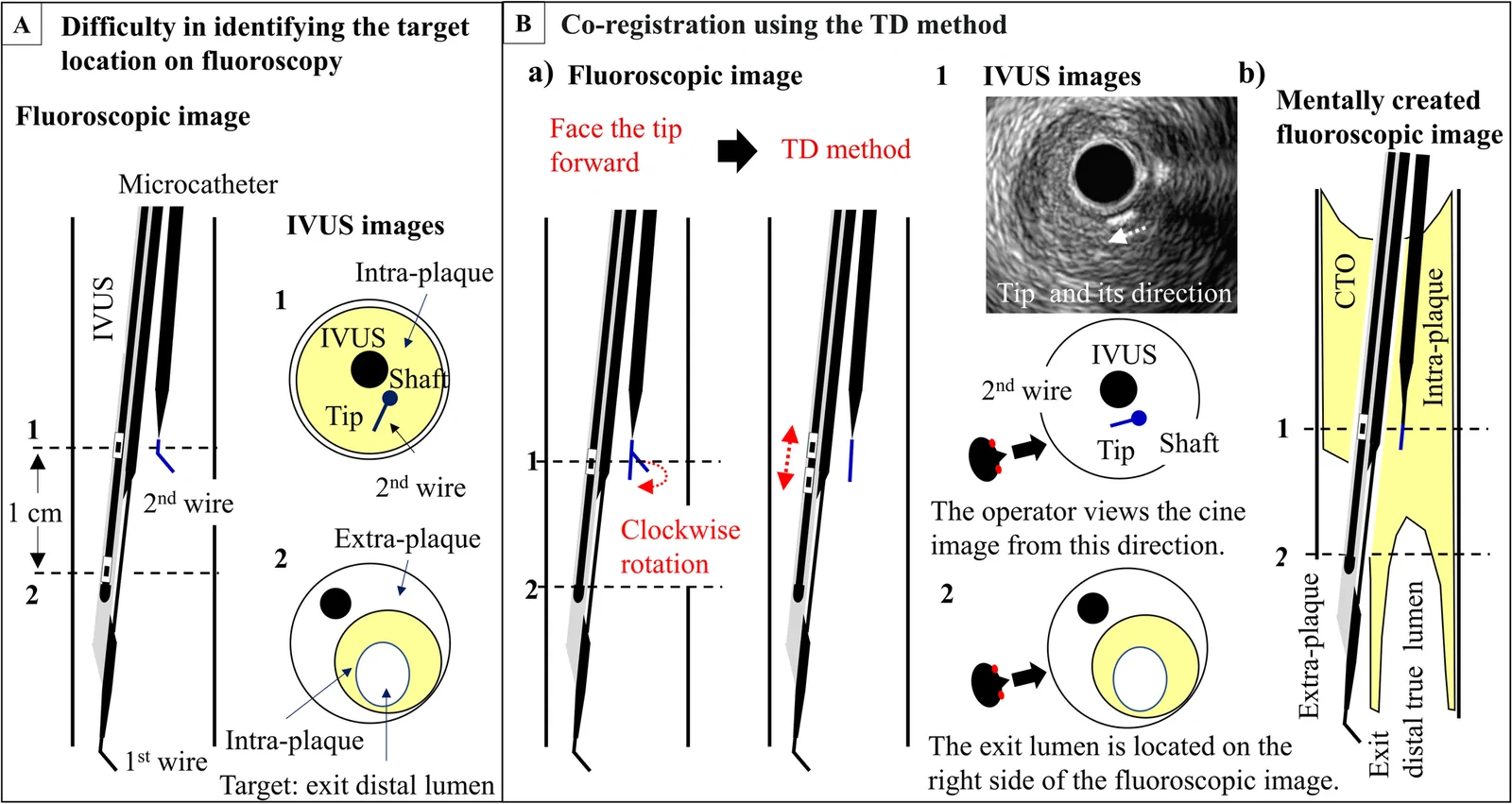
Co-registration of IVUS and fluoroscopic images using the TD method. (**A**) Schematic of a fluoroscopic view with corresponding numbered IVUS cross-sections. (**B**) (a) Schematics of fluoroscopic views with corresponding schematic IVUS images and an actual IVUS image at the numbered sites. (**B**) (b) A fused illustration of fluoroscopy and IVUS from a fluoroscopic perspective. *CTO* chronic total occlusion, *IVUS* intravascular ultrasound, *TD* tip detection

Before performing IVUS-guided wiring, fusing (co-registering) the IVUS image with the fluoroscopic image enables 3D wiring under 3D visualization not only on the IVUS image but also on the fluoroscopic image [22]. Figure 10 explains the procedure for identifying the location of the distal true lumen (DTL, target) on the fluoroscopic view. Previously, this fusion was performed by rotating the C-arm to visualize the spatial relationship between the IVUS transducer and the guidewire; however, this approach lacked sufficient precision [3]. Therefore, it can now be performed more simply using the TD method [22].

Specifically, based on the fluoroscopic image alone (Fig. 10A, left), the orientation of the target (DTL) as seen on the IVUS image is unknown. To address this, the tip of the second guidewire is oriented toward the operator at a point approximately 1–2 cm proximal to the final target. The TD method is then performed with IVUS to visualize this tip (Fig. 10Ba). This reveals that the fluoroscopic viewing direction corresponds to the 8 o’clock position on the IVUS monitor and consequently, the target located 1–2 cm distally is understood to be on the right side and slightly posterior on the fluoroscopic monitor (Fig. 10Bb). Recently, Fukata, Yoshikawa et al. reported that the COA view (Calculation of the Optimal Angiographic view using IVUS) method enables more precise fluoroscopic manipulation by identifying the optimal detector angle that provides the clearest separation between the IVUS catheter and the target [26].

**Fig. 11:**
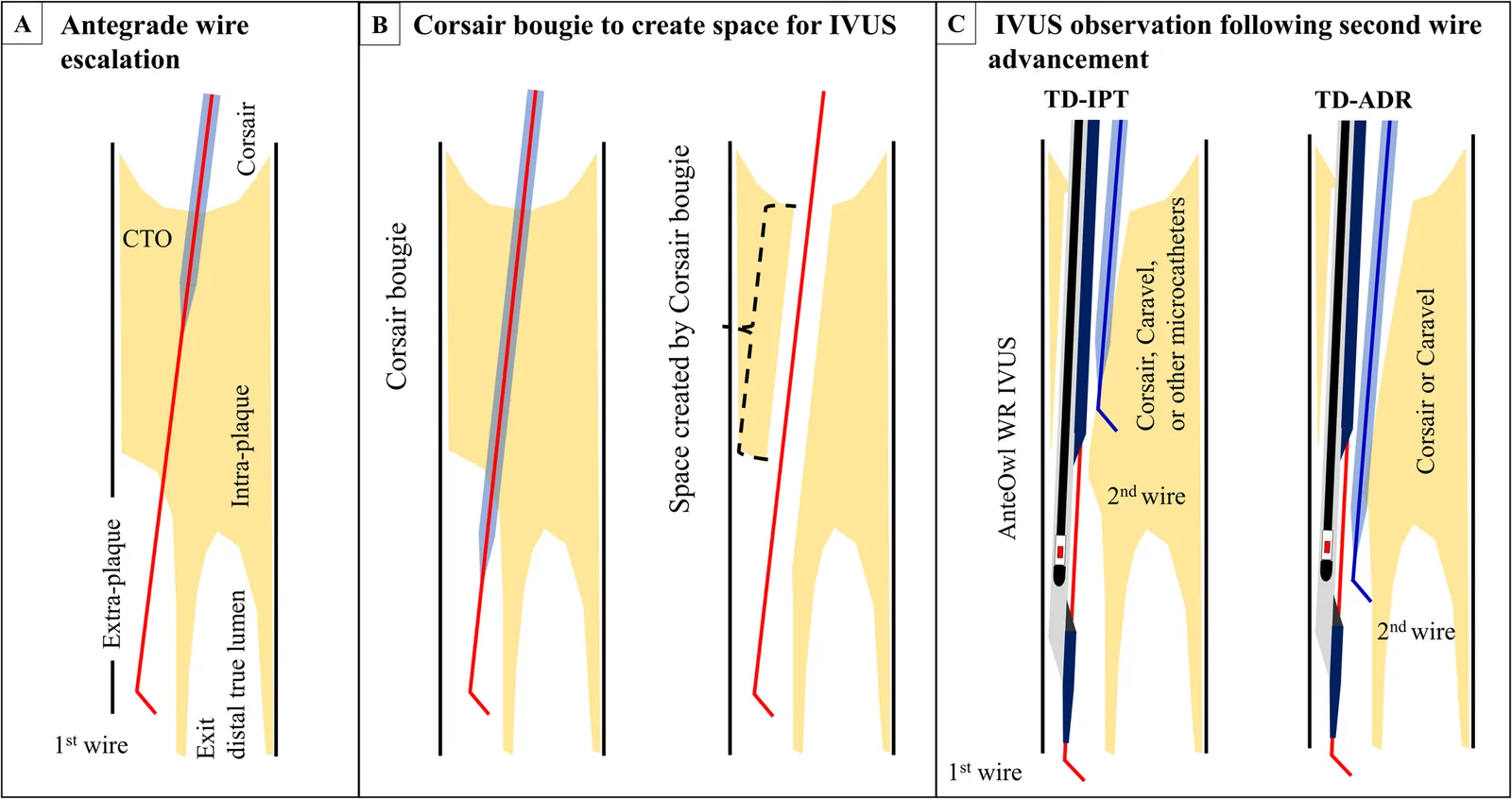
Procedural flows prior to IVUS-guided wiring. (**A–C**) Schematics of the fluoroscopic views leading up to the TD method. *TD* tip detection

While it is possible to insert a Corsair microcatheter and an AnteOwl WR through a 7 Fr guiding catheter, an 8 Fr guiding catheter is preferable for procedural stability as it can accommodate three devices: a Caravel microcatheter, an AnteOwl WR, and an anchor balloon. Figure 11 explains the procedure up to the point of observation inside the CTO lesion using the AnteOwl WR. First, AWE is performed (Fig. 11A). If the first guidewire is suspected of having entered an extra-plaque space, a Corsair is advanced to create a passage (bougie) to allow for the advancement of the IVUS catheter (Fig. 11B). Subsequently, the first guidewire is exchanged for a 0.014-inch intermediate stiff wire, such as the Ultimate Bros 3 (Asahi Intecc) or Gladius EX, via the Corsair. Prior to IVUS observation of the CTO lesion, a second guidewire (TD-IPT: Conquest Pro 20 g or CP12ST; TD-ADR: CP12ST) is advanced over the first guidewire using a dual-lumen catheter (DLC). To prevent air introduction and unnecessary pressure on the CTO tissue, the second guidewire should be advanced through the dual-lumen catheter (DLC) while the catheter is either outside the body or positioned 5 mm proximal to the CTO entry point. The second guidewire tip should be advanced until it is just proximal to the exit of the OTW lumen. The DLC is then advanced into the CTO lesion, and the second guidewire is initially advanced from the OTW lumen at the predicted TD site to minimize expansion of the extra-plaque space. A trapper device is then used to advance the Corsair through this second guidewire to a position just before the target inside the CTO. When a lower-profile microcatheter is required, various microcatheters are suitable for TD-IPT because they are stabilized by the surrounding CTO tissue. In contrast, the Caravel is preferred for TD-ADR due to its superior backup support, which remains effective even within the “open” extra-plaque space. Finally, the AnteOwl WR is advanced over the first guidewire to a position where the target can be visualized. The pullback function is utilized to observe the inside of the CTO, allowing for the selection of either a TD-IPT or a TD-ADR strategy (Fig. 11C). Proceed directly to second-wire manipulation using the TD method. However, if the advancement of the Corsair is difficult, the CTO lesion should be dilated with a 1.5 mm to 2.0 mm balloon, after which the subsequent steps are performed in the same manner.

## IVUS-based 3D wiring: selection between TD-IPT and TD-ADR

**Fig. 12:**
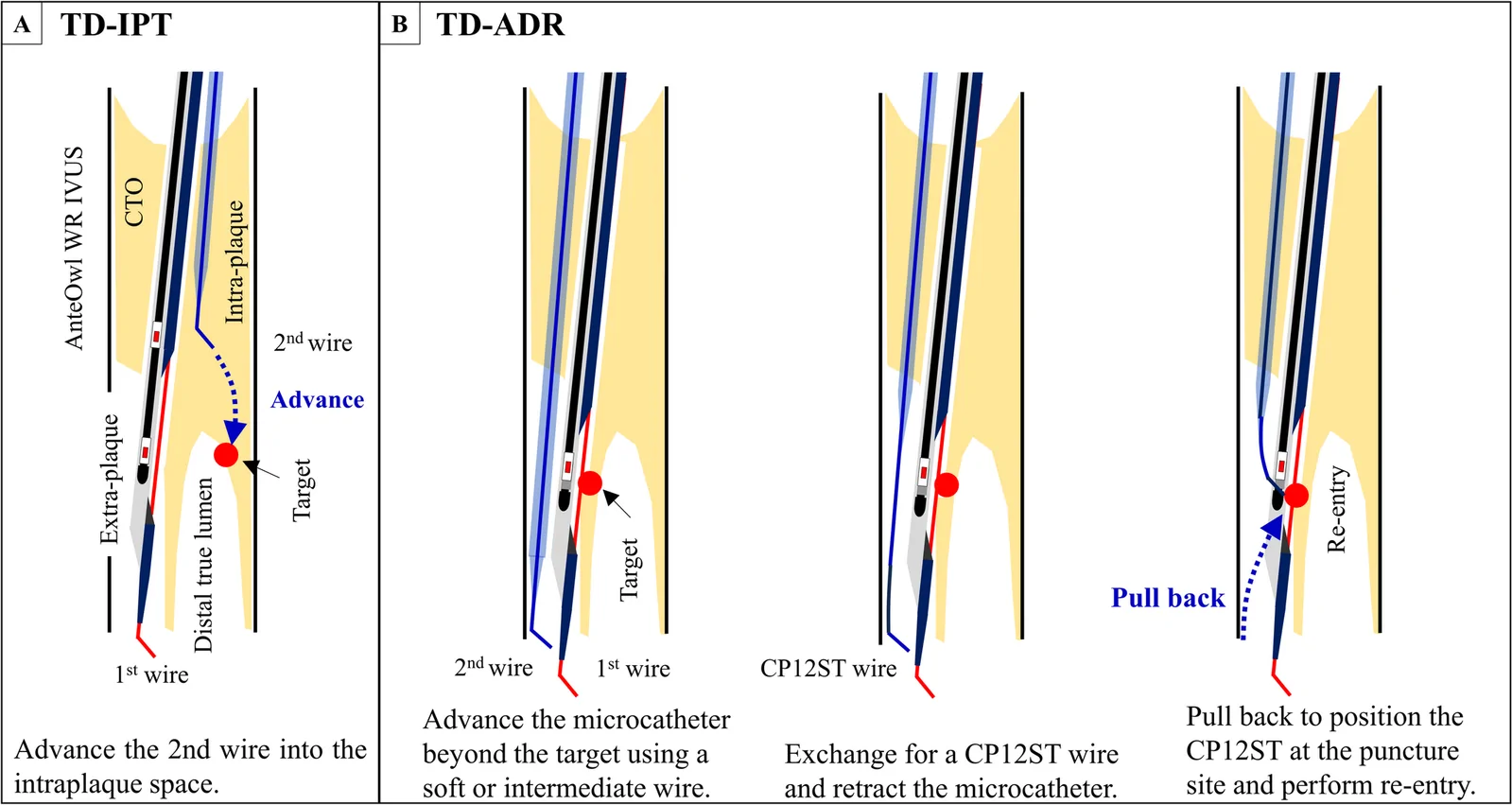
Distinct wiring approaches in TD-IPT vs. TD-ADR. (**A**,** B**) Fluoroscopic schematics depicting the wire manipulation in each method. *CTO* chronic total occlusion, *CP12ST* Conquest Pro 12ST, *IVUS* intravascular ultrasound, *TD-IPT* tip detection-intraplaque tracking, *TD-ADR* tip detection-antegrade dissection re-entry

TD-IPT is advantageous for lesion modification and side branch preservation because it dilates the lesion from inside the plaque. On the other hand, TD-IPT requires navigating the second guidewire from before the target (Fig. 12A). Conversely, TD-ADR offers a simpler approach, involving microcatheter advancement beyond the re-entry site followed by CP12ST wire insertion. Re-entry is then achieved by withdrawing the guidewire and puncturing at the designated target location (Fig. 12B). In summary, TD-IPT is preferable from the standpoint of achieving appropriate lesion dilatation, while TD-ADR is desirable for its technical simplicity. For this reason, the initial approach is to attempt TD-IPT, provided there are no obstacles to guidewire passage, such as severe calcification or extreme tortuosity. If TD-IPT fails to cross the CTO lesion, or if TD-IPT is anticipated to be difficult, the strategy is switched to TD-ADR. Particularly when the distal cap is located at a bifurcation, crossing via intra-plaque tracking using TD-IPT is preferred. However, if this proves difficult, consider using TD-ADR to achieve re-entry into the CTO body (TD-ADR to the CTO body) [27], or to perform precise pinpoint TD-ADR at the bifurcation.

## Practical guidewire manipulation in TD-IPT

**Fig. 13:**
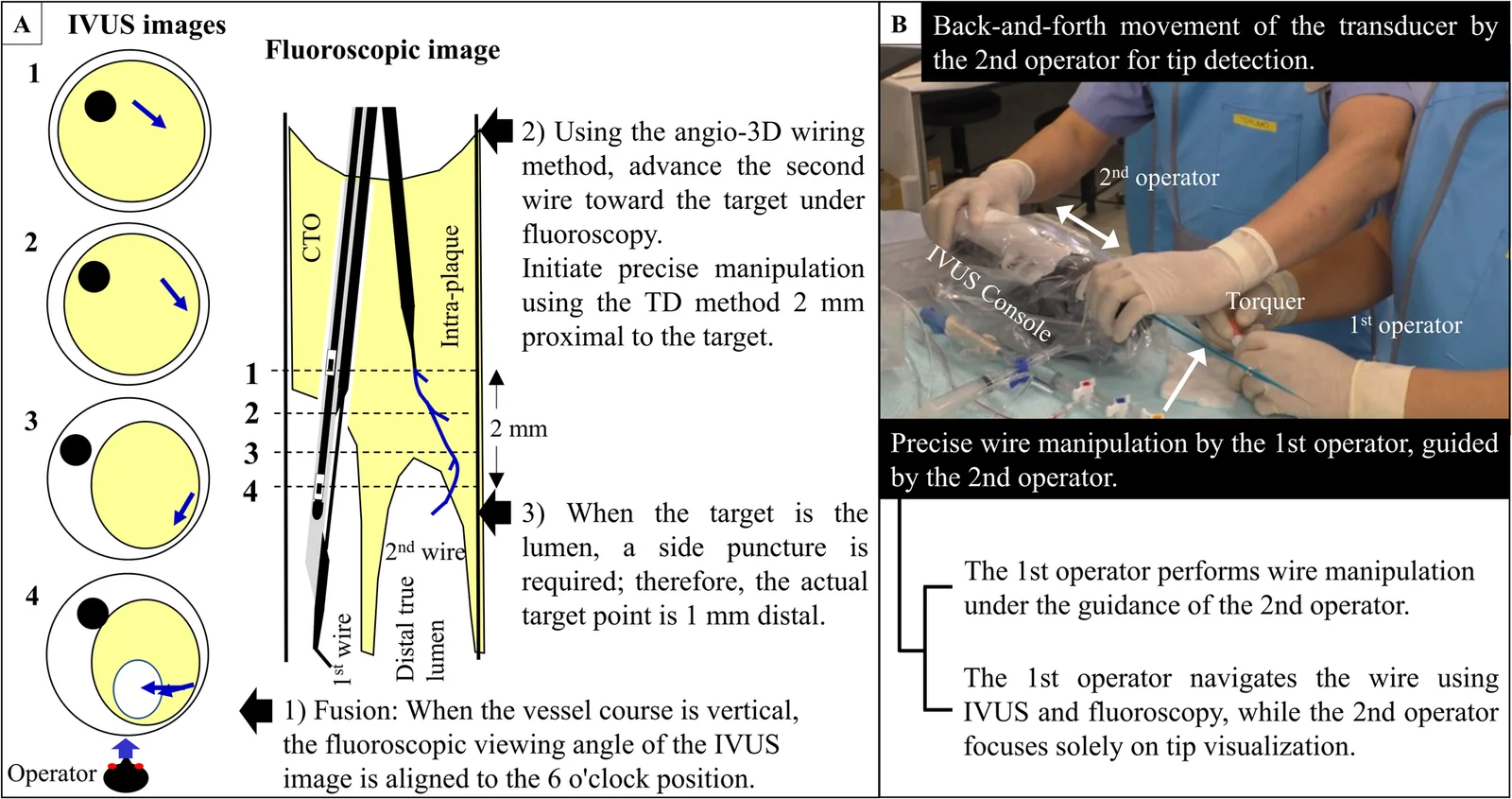
Wire navigation method in TD-IPT. (**A**) Schematic of a fluoroscopic view with corresponding schematic IVUS images at the numbered sites. 1), 2), and 3) indicate the order of the procedure. (**B**) Photograph showing coordination between the first and second operators during the TD procedure

Figure 13 details the procedure, specifically highlighting the distinct roles of the first and second operators during the TD method. As shown in Fig. 13A, the first and second operators share information to determine the guidewire crossing route via IVUS imaging, starting 2–3 mm proximal to the final target. As previously described, the second operator performs repeated longitudinal scanning—from the base of the guidewire tip to its apex—ensuring that the tip vanishes from the ultrasound slice to confirm its precise position. This maneuver is referred to as the ‘tip-truncation’ (tip-kiri) technique. During the TD method, the guidewire tip can be lost due to the movement caused by the first operator’s manipulation. Therefore, visualizing the tip in real-time synchronization with the guidewire’s movement requires a certain degree of training. The second operator uses IVUS imaging to guide the guidewire rotation. Meanwhile, the first operator—identifying the target mainly via fluoroscopy—performs the fluoroscopic 3D wiring technique to visualize the longitudinal axis and advances the guidewire with a precise 5–10-degree rotation. The second operator takes the lead, directing the guidewire manipulation while visualizing the tip in real-time, synchronized with the guidewire’s movement. However, as illustrated in Fig. 13B, a situation where two operators are both experts in the TD method is rarely encountered. Even if only one expert TD operator is present, provided the second operator focuses solely on visualizing the guidewire tip (and the target, if necessary), it is possible for the first operator—with sufficient training—to independently perform guidewire manipulation by integrating longitudinal information derived from the IVUS images demonstrated by the second operator.

**Fig. 14:**
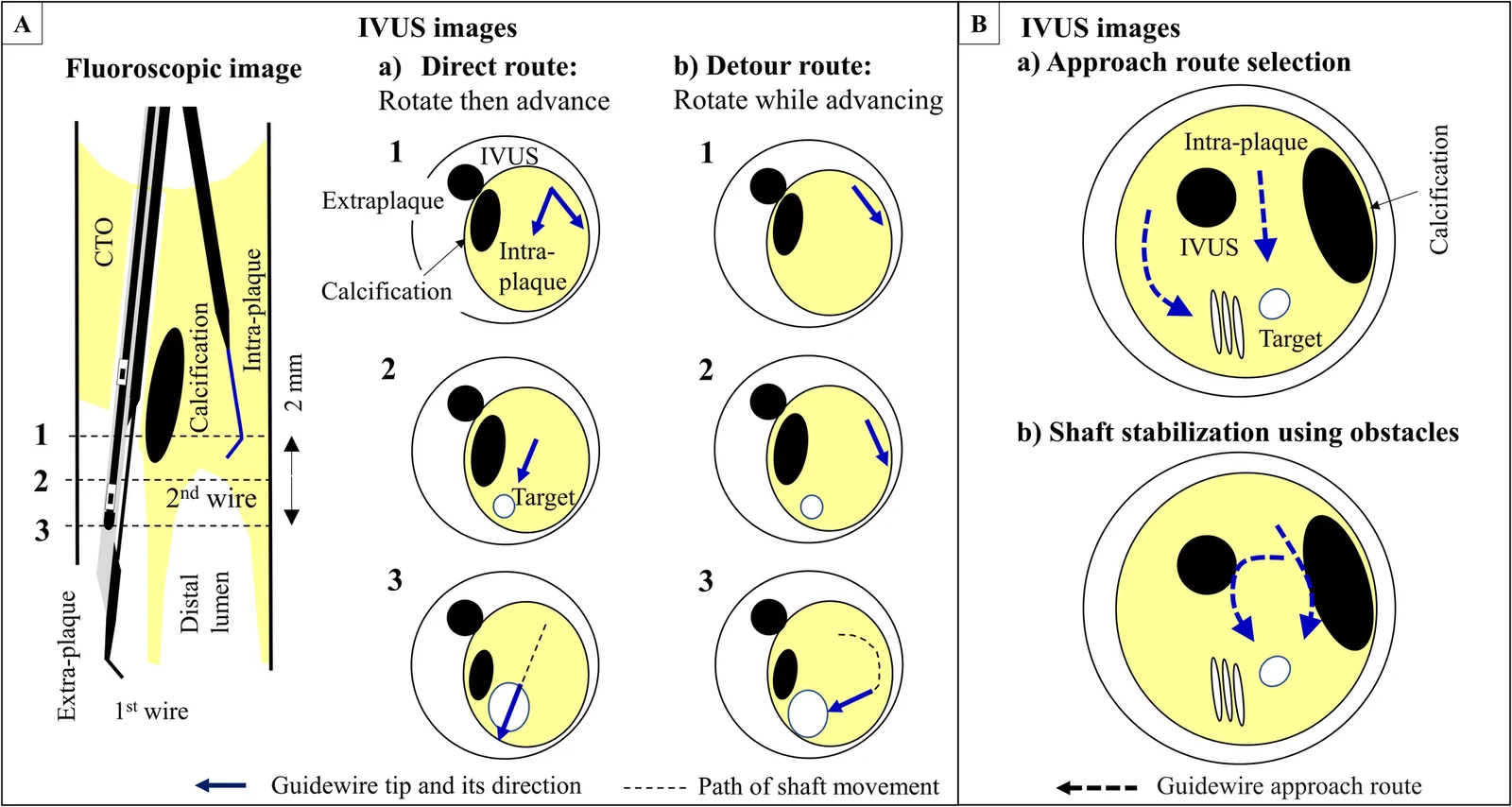
The detour theory and utilization of obstacles during TD-IPT. (**A**) Schematic of a fluoroscopic view with corresponding schematic IVUS images at the numbered sites. (**B**) Schematic illustration of an IVUS image showing the utilization of intraplaque obstacles. *CTO* chronic total occlusion, *IVUS* intravascular ultrasound, *TD-IPT* tip detection-intraplaque tracking

Advancing a guidewire inside a CTO lesion requires an understanding of the “Detour Theory” and how to manage obstacles such as the IVUS catheter and calcifications. The Detour Theory was proposed by Nagai. When advancing a guidewire with a 1-mm, 45-degree tip longitudinally toward a target within a CTO lesion, it is necessary to control the travel distance in the short-axis direction. This principle is similar to an airplane landing. Figure 14Aa illustrates the maneuver without a detour. In this method, the tip is first directed toward the target and then pushed; however, the tip passes over the target, failing to penetrate it. Figure 14Ab shows the maneuver with a detour. By pushing the guidewire while rotating the tip, the guidewire follows a curved path to the right. This effectively shortens the travel distance to the target, allowing for successful penetration.

Figure 14B describes methods for utilizing obstacles such as the IVUS catheter and calcifications. Since the IVUS catheter divides the luminal space into two compartments, the operator must determine which space to approach from (Fig. 14Ba). Additionally, the operator should consider utilizing the IVUS catheter or calcifications to support the guidewire shaft, thereby increasing the guidewire’s penetration force (Fig. 14Bb).

## Discovery of TD-ADR and its advantages over Stingray-ADR

**Fig. 15:**
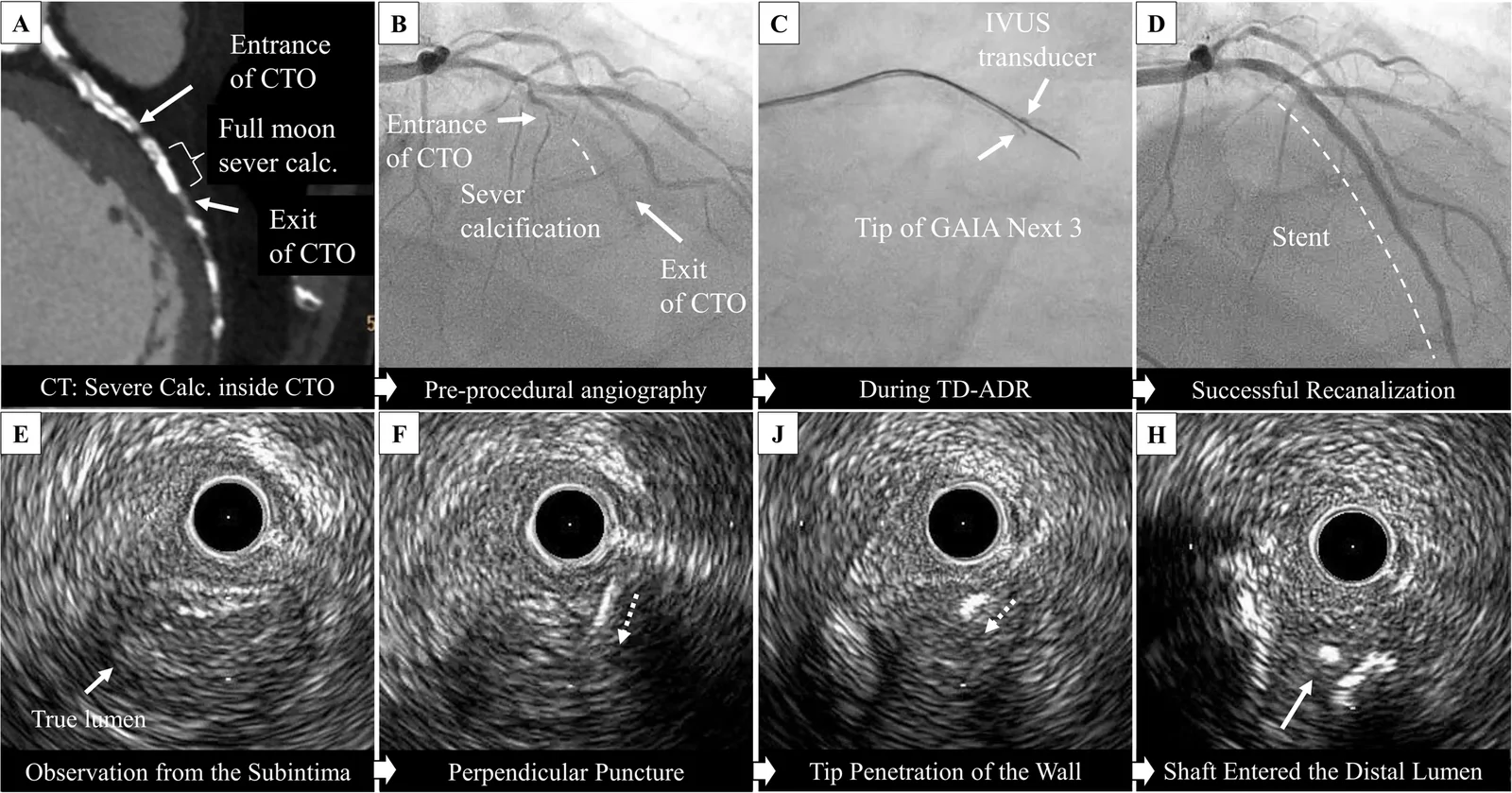
First TD-ADR Case. Computed tomographic image (**A**), angiographic images (**B**) prior to, **C** during, and **D** after the procedure, and intravascular ultrasound images **E** prior to, **F**, **G** during the puncture, and **H** after successful puncture. *CTO* chronic total occlusion, *IVUS* intravascular ultrasound, *TD-ADR* tip detection-antegrade dissection re-entry.

Conventionally, the Stingray system has been a prerequisite for performing standard ADR. The TD method was originally developed with the intention of intra-plaque tracking, and the discovery of TD-ADR was serendipitous. The first TD-ADR procedure was performed in November 2020, with Okamura as the first operator and Suzuki as the second operator [7]. In a case involving a severely calcified LAD CTO with a 20-year occlusion duration, we attempted Stingray-ADR from the extra-plaque space but failed to achieve re-entry. IVUS imaging from the extra-plaque space revealed the injury site on the DTL wall caused by the Stingray-wire penetration. We switched to the TD method to navigate the guidewire past this damaged area. However, during guidewire navigation with the TD method, the tip of the GAIA Next 3 wire (Asahi Intecc) was directed toward a healthy portion of the wall. Acting on instinct, we performed a perpendicular puncture, and re-entry was achieved (Fig. 15, ESM Video 3, Source: Reference 7). The procedure left us with a lingering sense of unease, and later that night Suzuki reviewed the IVUS images and confirmed that the guidewire had penetrated a normal section of the vessel wall. This finding led to the discovery of TD-ADR.

**Fig. 16:**
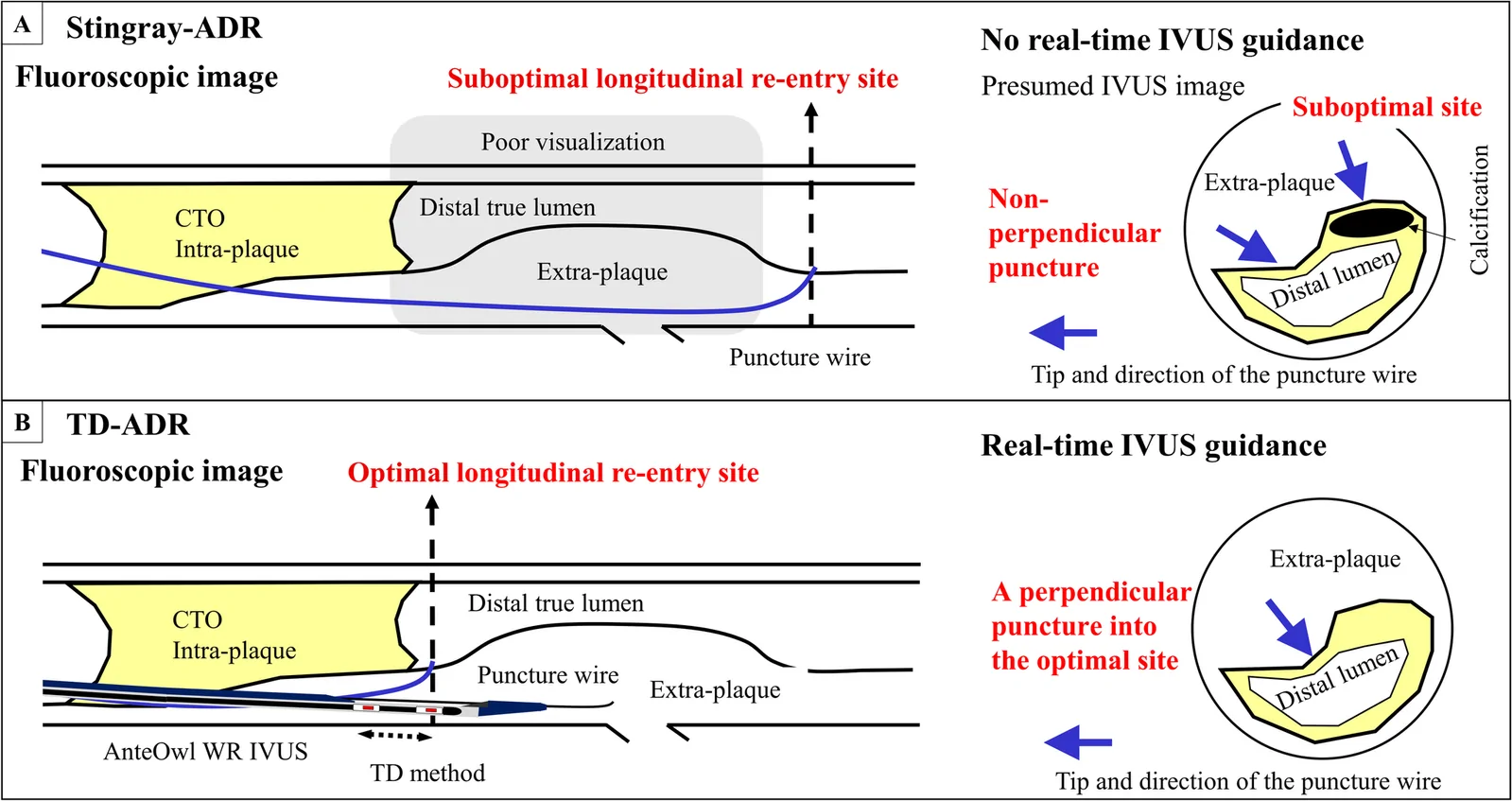
The pros and cons of Stingray-ADR and TD-ADR. (**A**,** B**) Schematic illustration of the fluoroscopic view and the IVUS image at the dotted line. *CTO* chronic total occlusion, *IVUS* intravascular ultrasound, *TD-ADR* tip detection-antegrade dissection re-entry. *Stingray-ADR* Stingray-antegrade dissection re-entry

The Stingray-ADR is an angiography-guided re-entry technique, and it faces problems with visualizing the puncture site, which necessitates shifting the puncture to a more distal location. Furthermore, detailed evaluation of the shape of the puncture site and perpendicular puncture with the guidewire at the re-entry point are also impossible. Consequently, even with pre-procedural case selection, its success rate is only around 60–80% [28]. TD-ADR is IVUS-guided, allowing for clear visualization of the puncture site and enabling perpendicular puncture at the optimal site, thus making highly accurate re-entry possible (Fig. 16) [8, 14]. Moreover, at the end of 2020, the CP12ST became available [17], which accelerated the standardization of TD-ADR. The CP12ST facilitates puncture irrespective of the thickness of the tissue separating the extra-plaque and intra-plaque spaces. This capability enables penetration into the CTO body—specifically TD-ADR to the CTO body—which was previously unachievable with conventional ADR techniques [28].

## TD-ADR: understanding unique wire movement and steps toward puncture

Guidewire movement in TD-ADR is different from standard CTO wiring. In TD-IPT, the movement is a “pivot” where the shaft is fixed in the plaque and only the tip turns. However, during TD-ADR, the guidewire is in the extra-plaque space (an open space), so the shaft is not fixed and moves together with the tip. Therefore, evaluating the “bias” of the second guidewire relative to the intra-plaque area (e.g., DTL) via IVUS imaging is essential when selecting the optimal puncture plane along the extra-plaque track. Additionally, shaft stabilization using the “six-point approach” is critical, even within the selected puncture plane [29].

As stated above, if wiring under fluoroscopy is unsuccessful and IVUS-guided wiring is considered, a dual-lumen microcatheter should be used first to advance the second guidewire along with its microcatheter. Subsequently, IVUS observation is performed over the first guidewire, followed by an immediate transition to the TD method. Regarding the choice of the second guidewire and its advancement, if there is a high likelihood of performing TD-ADR from the outset, a CP12ST should be advanced beyond the re-entry point predicted by fluoroscopy. For pre-dilation of the proximal lesion, the Corsair bougie technique is typically the first choice. However, if device delivery is expected to be difficult—particularly in cases with severe proximal calcification—firm dilation should be performed using an approximately 2.0 mm balloon. Notably, in TD-ADR, re-entry wire crossing remains feasible even if the target distal true lumen or intra-plaque area is collapsed due to proximal lesion dilation—in contrast to TD-IPT, where intra-plaque wiring becomes significantly more challenging.

Proper microcatheter selection is vital for TD-ADR, and high-support metal microcatheters like Corsair or Caravel are recommended. Unlike TD-IPT, where the catheter is fixed by the plaque, the microcatheter in TD-ADR sits in the “open” extra-plaque space and is not stabilized. Therefore, the catheter’s own support strength becomes essential. If you are already using a Corsair for AWE, you can typically continue with it. However, if you are using an 8 Fr guiding catheter and planning an anchor balloon technique, switching to a Caravel is recommended; its low profile accommodates both the anchor balloon and the IVUS catheter simultaneously within the 8 Fr guiding catheter.

## TD-ADR: determining the re-entry plane and puncture approaches

**Fig. 17:**
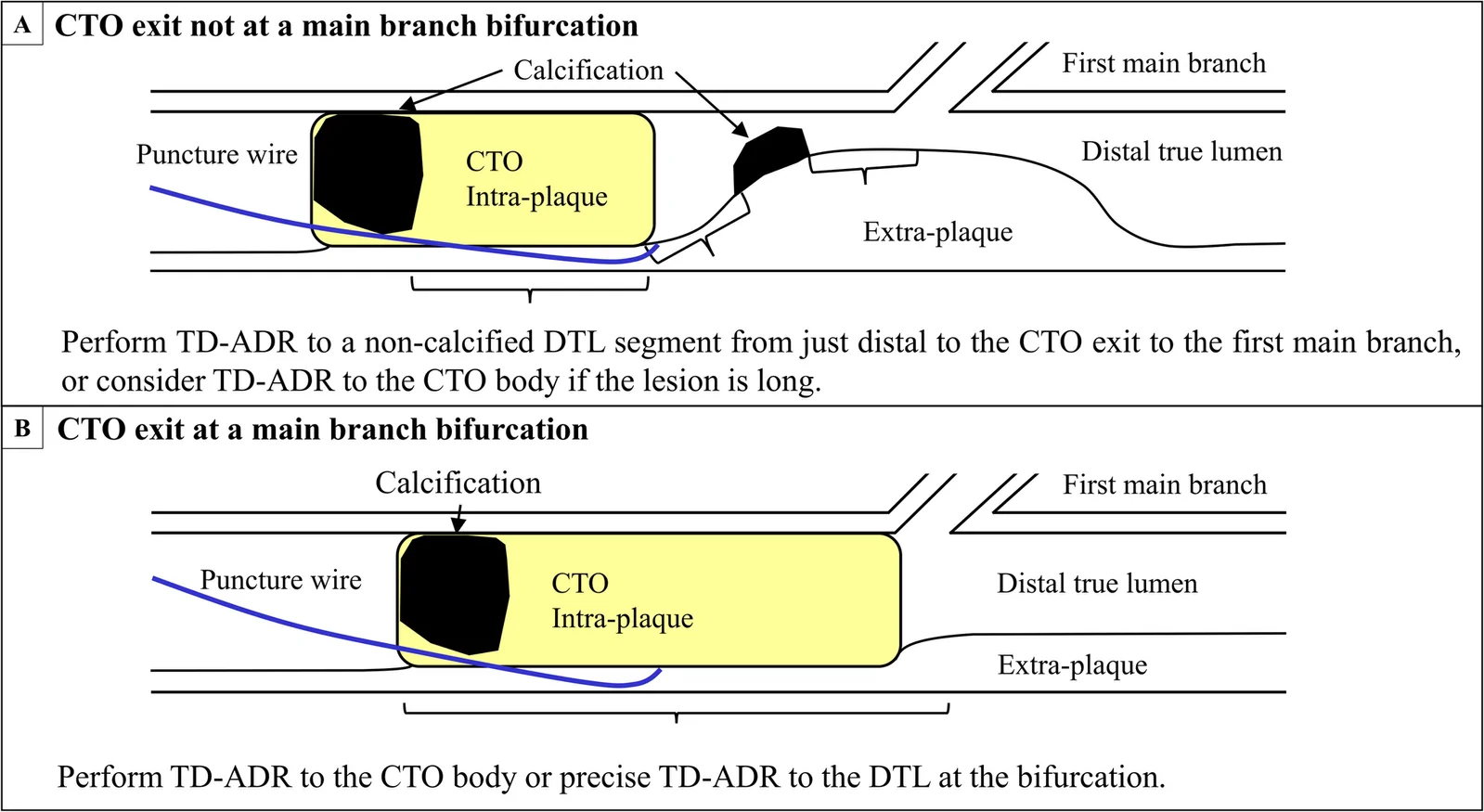
Long-axis view: Recommended Puncture Sites. Schematic illustration of the fluoroscopic view. *CTO* chronic total occlusion, *TD-ADR* tip detection-antegrade dissection re-entry, *DTL* distal true lumen

First, we describe the selection of the puncture site in the longitudinal view for TD-ADR. TD-ADR is categorized into two types: TD-ADR to the CTO body and TD-ADR to the DTL. If there is no risk of occluding significant side branches, TD-ADR to the DTL is preferred due to its procedural simplicity. Candidate puncture sites should be non-calcified segments located between the immediate CTO exit and the first major side branch, with priority given to sites closer to the exit (Fig. 17A). Furthermore, when the CTO exit is located at a bifurcation, puncture at the bifurcation itself is a potential option. However, this approach is technically challenging as it requires a precise, single-point puncture without any longitudinal deviation. Consequently, TD-ADR to the CTO body should also be considered, aiming for re-entry within the plaque proximal to the side branch (Fig. 17B). Thus, one of the primary advantages of TD-ADR over Stingray-ADR is the ability to achieve re-entry within the CTO body, which was previously technically impossible.

**Fig. 18:**
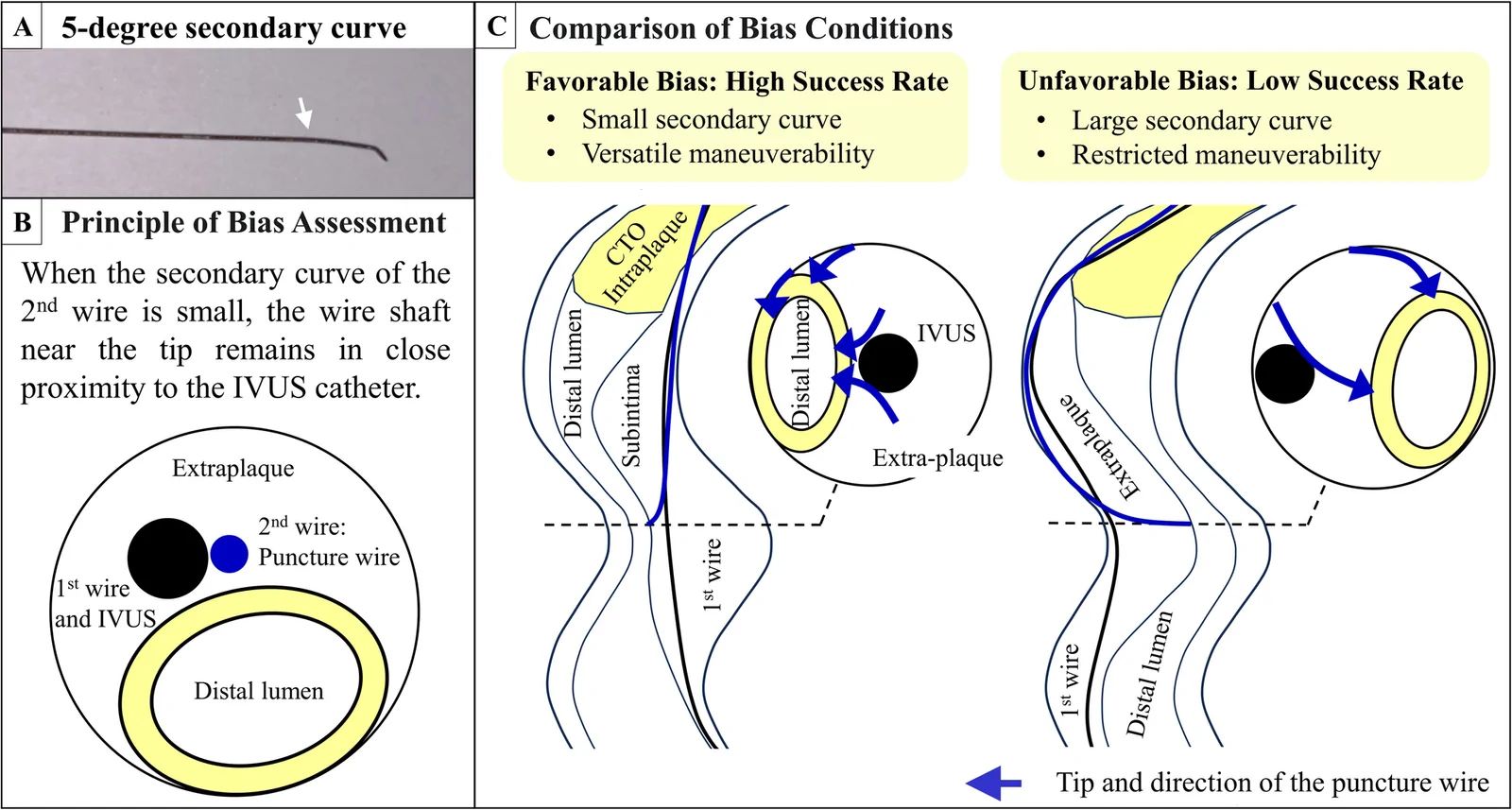
(**A**) Shaping of the re-entry wire with a 5-degree secondary curve. (**B**) Schematic illustration of the principle of bias assessment. (**C**) Comparison of bias conditions: schematic longitudinal fluoroscopic views and corresponding cross-sectional IVUS images at the plane indicated by the dotted line. *CTO* chronic total occlusion, *IVUS* intravascular ultrasound, *TD-ADR* tip detection-antegrade dissection re-entry

Next, we describe the selection of the puncture plane by evaluating the bias of the second wire relative to the target intra-plaque area, including the DTL. The bias of the second wire is assessed by the spatial relationship between the IVUS transducer and the intra-plaque area. Typically, in TD-ADR, a mild secondary curve of approximately 5 degrees is recommended to maintain both guidewire maneuverability and penetrating power (Fig. 18A). When the secondary curve of the guidewire is minimal, the shaft of the second wire is naturally guided close to the IVUS transducer (Fig. 18B). Therefore, by selecting a short-axis plane with “good bias”—where the IVUS transducer is positioned near the target intra-plaque area (empirically < 1 mm, which is approximately the diameter of the IVUS catheter, although further validation is required)—puncture can be achieved with a smaller secondary curve. A smaller curve enhances maneuverability, facilitating easier adjustments of the puncture site, including the “six-point approach” discussed later (Fig. 18C, left). In contrast, a plane with “poor bias,” where the IVUS transducer is distant from the target intra-plaque area, requires a larger secondary curve. However, this large curve limits the operator’s ability to adjust the puncture site freely. Moreover, the guidewire may easily bend at the secondary curve during the puncture attempt, which hinders successful penetration due to a loss of pushability (Fig. 18C, right). Nevertheless, as successful re-entry remains a possibility, this plane should be attempted if no better options are available.

**Fig. 19:**
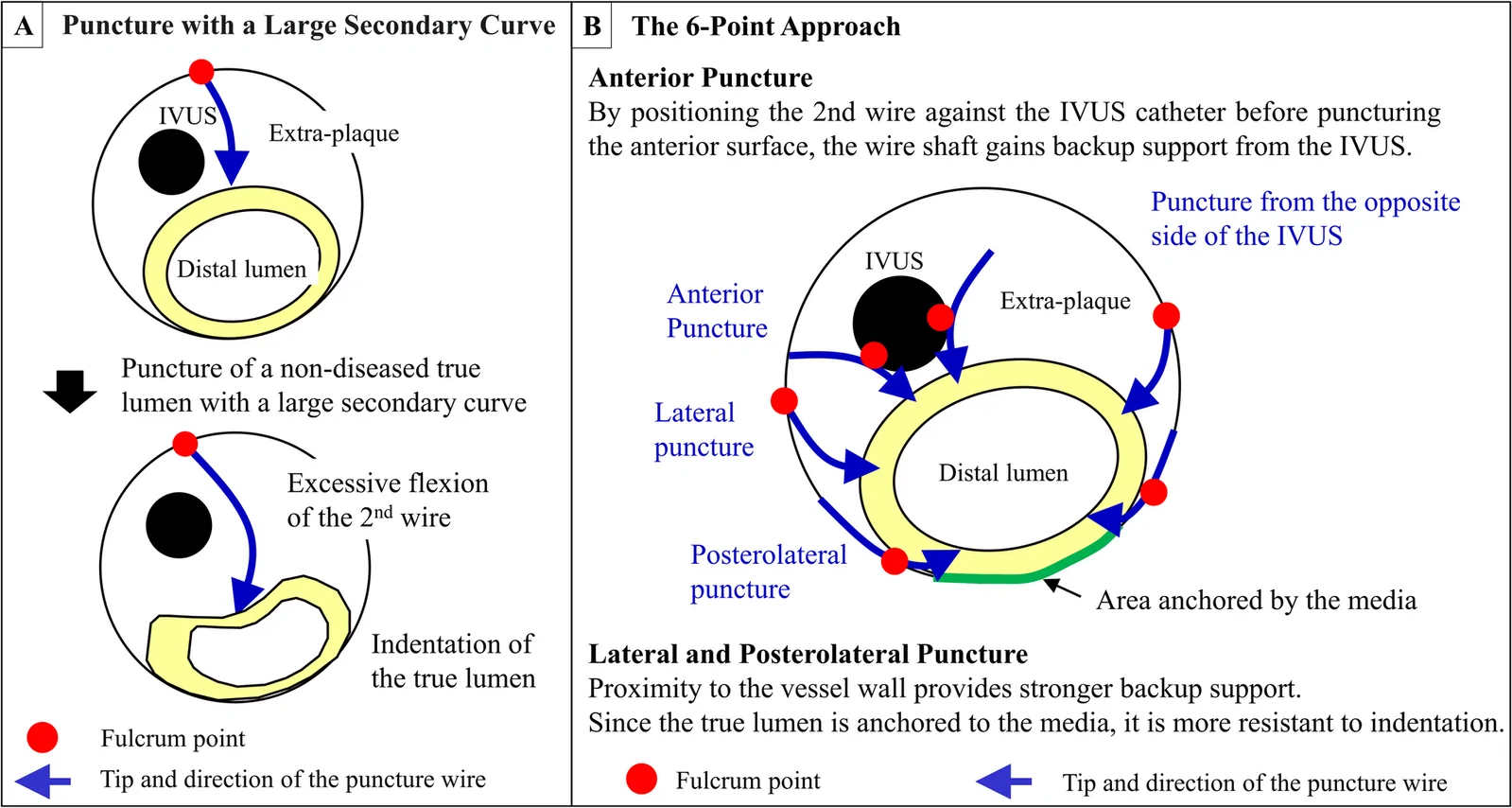
Determining the re-entry site in the short axis for TD-ADR: a mechanical perspective. (**A**,** B**) Schematic illustrations of IVUS images during re-entry in TD-ADR. *IVUS* intravascular ultrasound, *TD-ADR* tip detection-antegrade dissection re-entry

The following section describes the puncture site in the short-axis view. Similar to ultrasound-guided puncture of superficial vessels, deformation of the wall within the intra-plaque area occurs, particularly when targeting a non-diseased DTL (Fig. 19A). Consequently, the following six approaches are recommended. These involve selecting the direction of the puncture: “Anterior Puncture,” where the guidewire punctures from the front while utilizing the IVUS shaft for back-up support; “Lateral Puncture,” which facilitates shaft support from the vessel wall; and “Posterolateral Puncture” (Fig. 19B, ESM Video 4–7). In lateral and posterolateral punctures, the fixation of the media against the intra-plaque area results in less deformation of the wall, thereby increasing the probability of a successful puncture. Also, puncturing from the side opposite the IVUS should also be considered. Puncturing from the opposite side when the second wire is difficult to reach due to bias can be effective. Once the second wire is advanced to the opposite side, a force is generated that biases it back toward its original position, which can enhance its penetration power.

The CP12ST is pre-shaped with a 1.3 mm, 45-degree primary curve. As mentioned above, a very slight secondary curve of approximately 5 degrees should be formed about 8 mm proximal to the tip (Fig. 18A). Initially, the puncture is attempted with this secondary curve, and additional shaping is applied to the secondary curve as needed based on IVUS findings. Recently, successful TD-ADR cases using OptiCross IVUS have been reported by Opolski et al. in Europe [24] and Li et al. in North America [25]. In both instances, re-entry was achieved via lateral puncture.

**Fig. 20:**
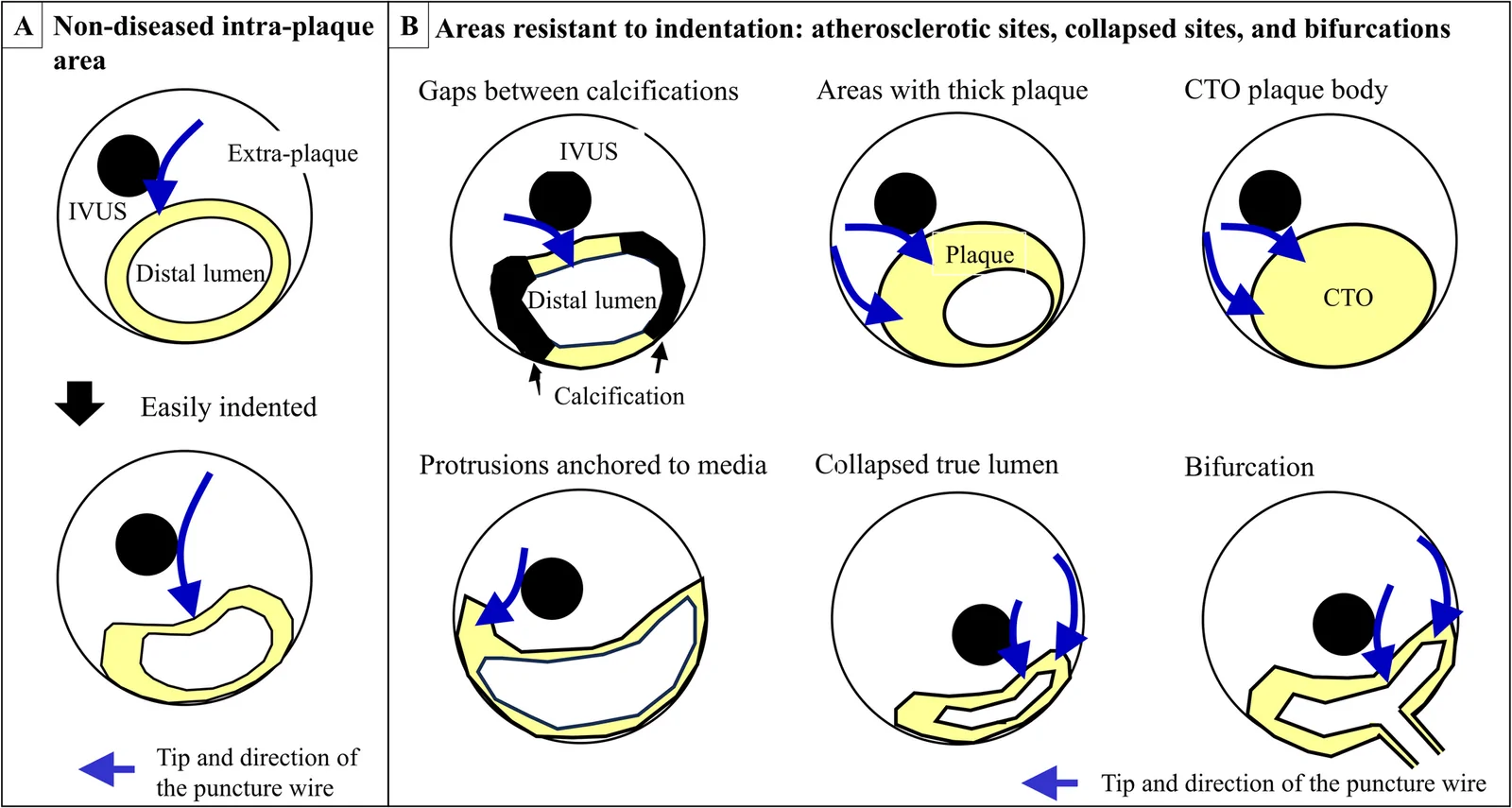
Determining the short-axis re-entry site in TD-ADR: lesion morphology perspective. (**A**,** B**) Schematic illustrations of IVUS images during re-entry in TD-ADR. *IVUS* intravascular ultrasound, *TD-ADR* tip detection-antegrade dissection re-entry

Furthermore, the morphology of the true lumen on IVUS imaging should be taken into account. As previously mentioned, a non-diseased intra-plaque area (DTL) is highly elastic and may undergo deformation during puncture (Fig. 20A). Potential candidate sites include calcified segments that provide accessible gaps for puncture, thick plaque regions including the CTO body, and collapsed areas that are resistant to further indentation (Fig. 20B).

## TD-ADR: practical steps for re-entry

**Fig. 21:**
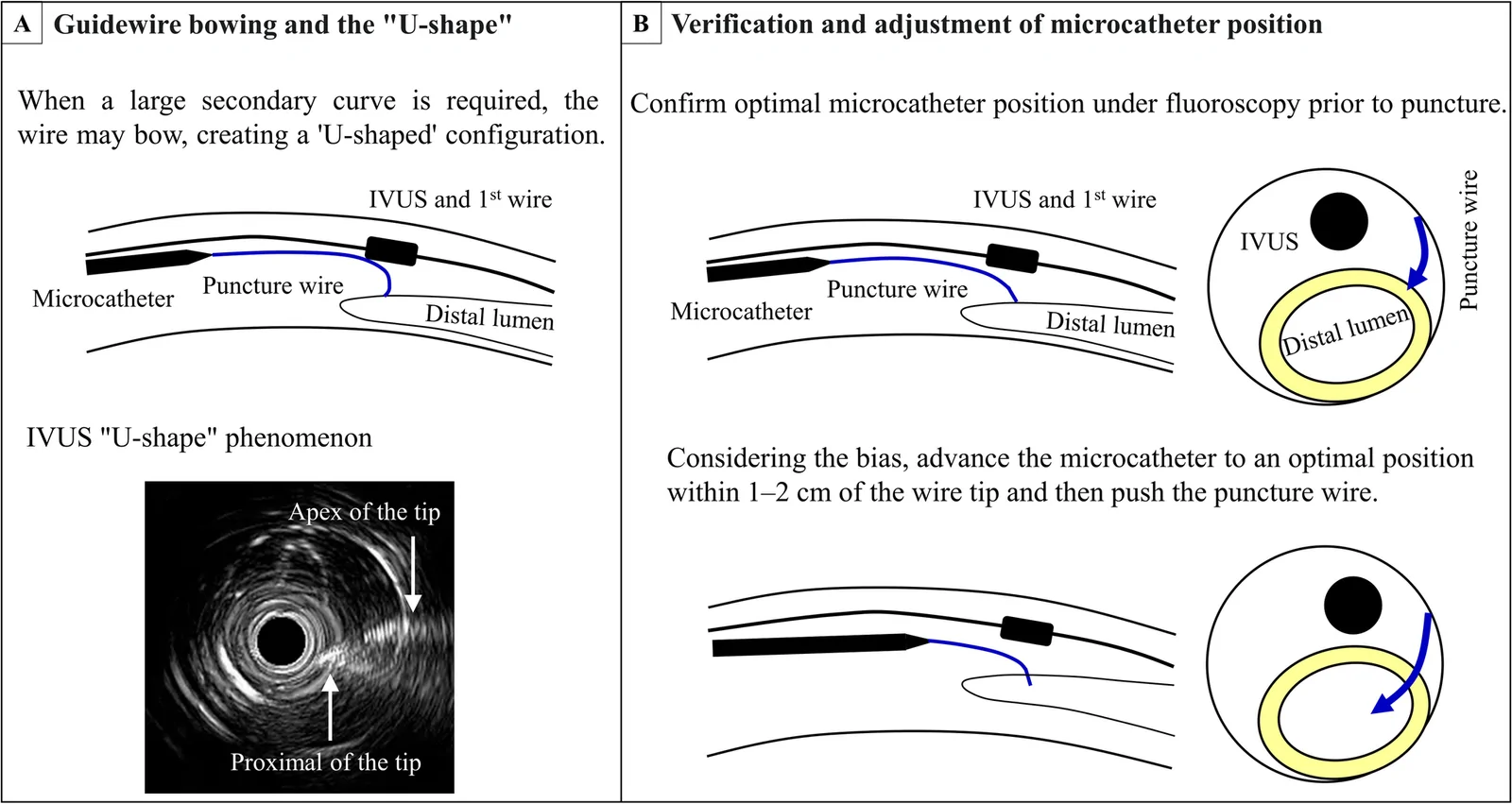
Advancing the microcatheter to the optimal position immediately prior to the final puncture in TD-ADR. (**A**) Schematic of fluoroscopic views and the actual IVUS image. (**B**) Schematic of fluoroscopic views and IVUS images during TD-ADR. *IVUS* intravascular ultrasound, *TD-ADR* tip detection-antegrade dissection re-entry

In the TD method, the guidewire tip is visualized to facilitate perpendicular puncture. After confirming that the tip has engaged the target surface, the microcatheter should be advanced under fluoroscopic guidance to a position 1–2 cm proximal to the tip to increase backup support before the final puncture (Fig. 21B). The decision of how far to advance the microcatheter—specifically, whether it should pass the secondary curve—should be made based on fluoroscopic findings. Puncture becomes difficult if the tip flexes into a “U-shape”. Kawamura (Kindai University Nara Hospital) proposed that this U-shaped deformation can be visualized using both fluoroscopy and IVUS (Fig. 21A). If the guidewire prolapses into a U-shape despite appropriate microcatheter advancement, the secondary curve should be reshaped (especially if it has a large angle exceeding 5 degrees) or an alternative puncture site should be selected.

**Fig. 22:**
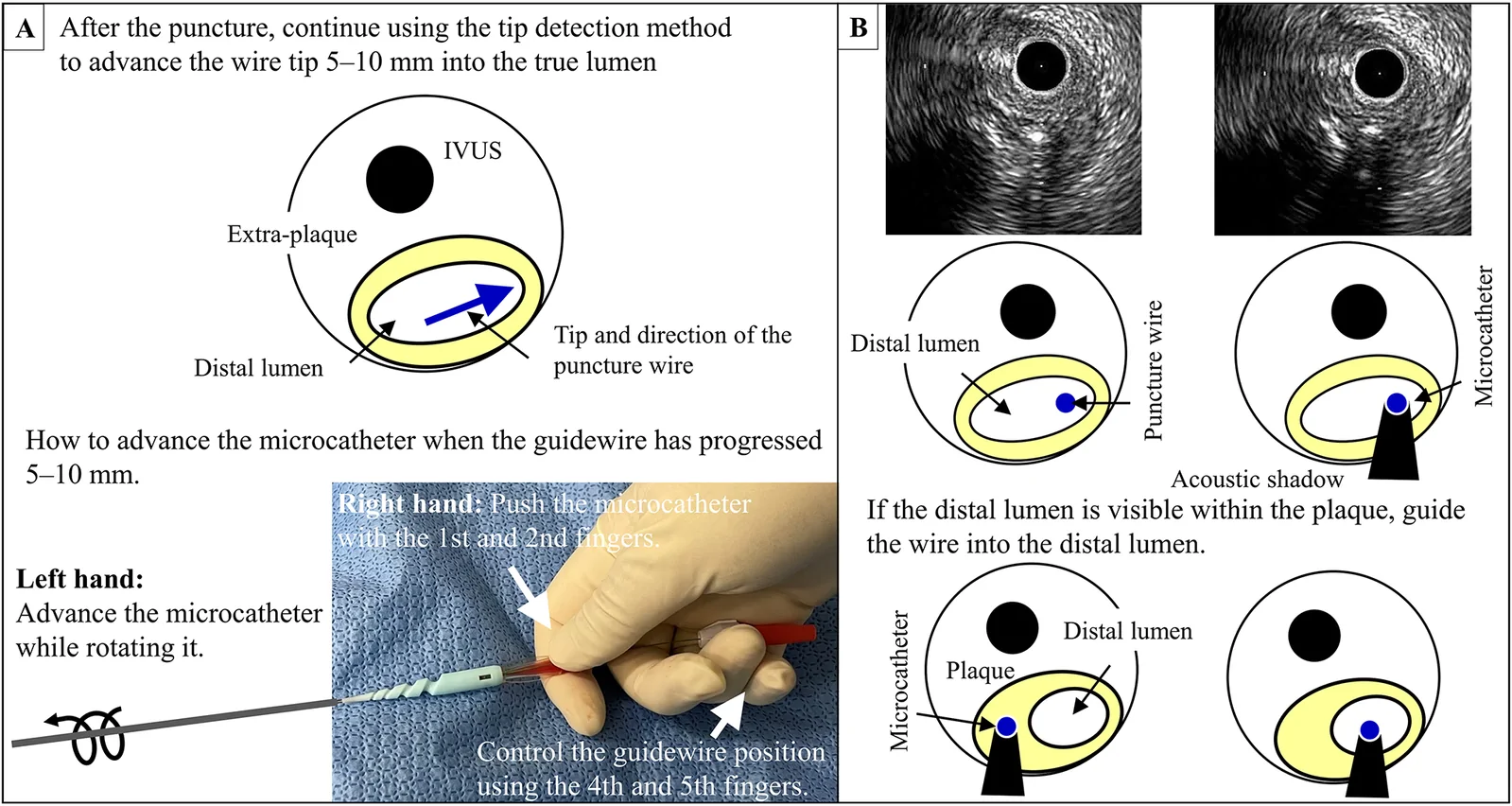
Advancing the microcatheter following puncture in TD-ADR　(**A**) Schematic illustration of the IVUS image and a photograph showing hand maneuvers for advancing the microcatheter. (**B**) Schematic illustration of the IVUS image and corresponding actual IVUS images. *IVUS* intravascular ultrasound, *TD-ADR* tip detection-antegrade dissection re-entry

Even after the guidewire tip enters the DTL, do not become complacent; continue the TD method to advance the guidewire at least an additional 5–10 mm within the DTL or the intra-plaque space (Fig. 22A). It should be noted that advancing a microcatheter over a guidewire that has only been advanced 5–10 mm past the puncture site requires a high degree of technical skill. It is crucial to advance the microcatheter using the left hand and the thumb and index finger (1st and 2nd digits) of the right hand, while simultaneously maintaining and controlling the guidewire’s position with the ring and little fingers (4th and 5th digits) of the right hand (Fig. 22A). It should be noted, however, that this technique is not unique to our method; it is a common practice among experienced PCI operators.

Once the guidewire tip is advanced distally, observe the guidewire shaft via IVUS at the re-entry point of the DTL to visually confirm that the tip of the microcatheter is being guided into the DTL. When the microcatheter reaches the DTL, it can be identified on the IVUS image by the acoustic shadowing (Fig. 22B). Subsequently, the CP12ST is withdrawn and exchanged for a soft wire to be advanced further distally. During re-entry with the CP12ST, if the DTL is small, the CP12ST may occasionally penetrate the DTL. However, this is not an issue as long as the CP12ST is advanced further and the microcatheter itself is maintained within the DTL under IVUS guidance. Additionally, if a high plaque burden is present but the DTL is visible, the CP12ST should be navigated to the DTL directly, followed by the advancement of the microcatheter. In the case of re-entry into the CTO body, after advancing the microcatheter, the CP12ST should be exchanged for a CTO tapered or stiff wire—rather than a soft wire—to resume CTO wiring (Fig. 22B, lower panel).

## Additional required for the TD method in CTO PCI: guidewire advancement and management of Hematoma

To perform the TD method, it is typically necessary to advance a guidewire antegradely into the CTO lesion. When conventional wiring fails to achieve antegrade advancement, various techniques such as knuckle wiring, Carlino, STAR [15], and BASE [16] become essential. Once the guidewire is successfully positioned within the CTO lesion, IVUS can be advanced to facilitate the TD method. Recently, HDR has attracted attention as a novel wire-crossing method that relies on contrast injection pressure rather than conventional guidewire manipulation [12]. In this technique, contrast medium is gently injected into the plaque to create a channel through hydraulic pressure; subsequently, a tapered soft polymer-jacketed wire with an unbent tip is advanced through this route. Similar to the Carlino and STAR techniques, HDR offers the advantage of visual vessel identification via contrast injection. At TCT 2025, Tadano and Fujita reported that the integration of the TD method and HDR enables a single-access approach without the need for contralateral injection, thereby reducing contrast volume and shortening procedure times while achieving high success rates. Their proposed strategy begins with an initial attempt at guidewire crossing using HDR. If the guidewire fails to cross, TD-ADR is performed to achieve definitive guidewire passage [13].

The TD method essentially involves advancing an IVUS catheter into the extra-plaque space. A major concern with this technique is the compromise of distal blood flow caused by the expansion of an extra-plaque hematoma. Current management strategies are as follows: (1) Creating a distal re-entry point using a cutting balloon [29, 30], and (2) Performing aspiration via a microcatheter positioned within the extra-plaque space [31]. The latter technique utilizes the first guidewire already advanced in the extra-plaque space before the TD-ADR procedure. After the second wire has been successfully navigated into the DTL, the microcatheter is advanced over the first guidewire as far distally as possible within the extra-plaque space. Following IVUS evaluation, a stent is deployed to adequately expand the lesion and seal the entry and re-entry points of the extra-plaque space. Aspiration through the microcatheter begins just before stent deployment and is sustained throughout the post-stenting period, including the entire withdrawal process of the catheter. In our clinical practice, we prioritize this aspiration method. If adequate distal flow is not restored, we then proceed to create a distal re-entry using a cutting balloon.

## Extended applications of 3D wiring and the TD method: expanding from CTO to open vessel PCI

In RWE for CTO PCI, guidewire crossing during Reverse CART can be challenging when the antegrade and retrograde wires are located in different compartments. This difficulty can be overcome by employing the TD method. A particularly challenging scenario occurs when the antegrade wire is in the extra-plaque space while the retrograde wire is in the intra-plaque space, as this often hinders successful connection via conventional Reverse CART. In such cases, the following “TD-assisted” strategies provide definitive solutions:

1. **TD-IPT**: Utilizing antegrade IVUS and the TD method to guide the retrograde wire successfully into the proximal true lumen. It should be noted that the IVUS orientation is reversed; therefore, the required clockwise and counter-clockwise torquing of the wire is inverted compared to standard antegrade wiring.
2. **TD-ADR**: Redirecting the antegrade wire from the extra-plaque space back into the intra-plaque space using the TD method, allowing for successful antegrade distal crossing.
3. **TD-assisted Reverse CART**: If the antegrade wire cannot be advanced distally even after re-entry, Reverse CART can be performed at a site where both guidewires are confirmed to be within the intra-plaque space, thereby facilitating a successful connection.

The 3D wiring technique is highly effective even in non-CTO (open vessel) lesions. As emphasized in the basic principles section, operators should consistently maintain torque-to-tip synchronization in open vessels to ensure a seamless transition to 3D wiring for both CTO and the following complex non-CTO cases. The following are specific scenarios for non-CTO 3D wiring:

1. **Difficult true lumen passage in acute myocardial infarction**: When a guidewire accidentally enters the extra-plaque space at the culprit lesion, the TD method can navigate the guidewire into the DTL [32].
2. **Acute coronary occlusion**: The TD method provides an effective bailout strategy for sudden occlusion of a main vessel or a side branch during PCI of non-CTO lesions [23, 33].
3. **Device-uncrossable calcified lesions**: For heavily calcified segments where devices fail to cross, the TD method can be used to intentionally create a bypass within the extra-plaque space to bypass the uncrossable segment.
4. **Side branch selection and crossing the optimal stent struts**: 3D wiring is an effective technique for targeting side branches [34]. Under fluoroscopy, the guidewire tip is advanced to the bifurcation. Once the depth is assessed, the guidewire is rotated from posterior to anterior; with an appropriate curve, the wire should engage the side branch. If unsuccessful, the wire tip should be reshaped, followed by the application of the TD method to precisely navigate into the side branch ostium. As noted by Suzuki, while the GAIA Next 1 (Asahi Intecc) is preferred for its superior torque response, the Miracle Neo 3 (Asahi Intecc) is a viable alternative due to its favorable safety profile and maneuverability. Furthermore, a Conquest Pro 9 g may be required for side branches jailed by stent struts. Yoshikawa also highlights the efficacy of this method for crossing the optimal stent strut when rewiring a jailed left circumflex artery following the left main trunk to the left anterior descending artery crossover stenting.
5. **Directional coronary atherectomy**: After determining the fluoroscopic projection for the directional coronary atherectomy, the TD method is utilized to co-register the IVUS-identified plaque location with the fluoroscopic view (Fig. 9) [35]. Furthermore, this co-registration is applicable to any coronary segment.

## Conclusions

While 3D wiring is fundamental to all PCI, its application in CTO cases elevates procedural precision significantly. Although initiating with TD-ADR is often preferable for time efficiency, progressive mastery of TD-IPT followed by angiography-based 3D wiring is essential to fully realize the potential of this approach. We look forward to the global availability of CTO-specific short-tip pullback IVUS and anticipate that 3D wiring techniques, including the TD method, will enhance CTO PCI success rates worldwide.

## Supplementary Information

Below is the link to the electronic supplementary material.

Video 1. Two-dimensional wiring using rotational ETOSS. Reproduced with permission from Okamura et al.

Video 2. Three-dimensional wiring based on the 3D imaging rule using rotational ETOSS. Reproduced with permission from Okamura et al.

Video 3. Intravascular ultrasound (IVUS) images showing distal true lumen puncture using the tip-detection method. Reproduced with permission from Okamura et al.

Video 4. Anterior puncture during tip detection-antegrade dissection and re-entry (TD-ADR)

Video 5. Anterior puncture into the gap between calcifications during tip detection-antegrade dissection and re-entry (TD-ADR)

Video 6. Lateral puncture during tip detection-antegrade dissection and re-entry (TD-ADR)

Video 7. Posterolateral puncture during tip detection-antegrade dissection and re-entry (TD-ADR). Case courtesy of Dr. Yoshikawa.s

## Abbreviations

2D: Two-dimensional
3D: Three- dimensional
ADR: Antegrade dissection and re-entry
AWE: Antegrade wire escalation
BASE: Balloon-assisted subintimal entry
CART: Controlled antegrade and retrograde tracking
CP12ST: Conquest Pro 12 sharpened tip
CTO: Chronic total occlusion
DTL: Distal true lumen
HDR: Hydrodynamic contrast recanalization
IVUS: Intravascular ultrasound
PCI: Percutaneous coronary intervention
RWE: Retrograde wire escalation
STAR: Subintimal tracking and re-entry
TD: Tip detection
TD-ADR: Tip detection- antegrade dissection and re-entry
TD-IPT: Tip detection-intraplaque tracking
