# Correction: Angiography- and IVUS-tip-detection-guided 3D wiring for CTO and non-CTO percutaneous coronary interventions

**DOI:** 10.1007/s12928-026-01313-y

**Published:** 2026-06-23

**Authors:** Atsunori Okamura, Ryohei Yoshikawa, Kota Tanaka, Satoshi Suzuki, Masato Ishikawa, Hiroyuki Nagai, Tomohiro Yamasaki, Kazunori Yamaji, Katsutoshi Kawamura, Naotaka Okamoto, Takayuki Ishihara, Wataru Nagamatsu, Takeshi Serikawa, Yoshiaki Ito, Heitaro Watanabe

**Affiliations:** 1 Cardiovascular Center, Sakurabashi Watanabe Advanced Healthcare Hospital, NAKANOSHIMA QROSS 4-3-51 Nakanoshima, Kita-ku, 530-0005 Osaka, Japan; 2 Department of Cardiovascular Medicine, Sanda City Hospital, Sanda, Japan; 3 Department of Cardiology, Kindai University Nara Hospital, Nara, Japan; 4 Department of Cardiology, Fujita Health University, Aichi, Japan; 5 Division of Cardiovascular Medicine, Department of Internal Medicine, Kurume University School of Medicine, Kurume, Japan; 6 Department of Radiology, Kindai University Nara Hospital, Nara, Japan; 7 Division of Cardiology, Osaka Rosai Hospital, Sakai, Osaka Japan; 8 Cardiovascular Center, Kansai Rosai Hospital, Amagasaki, Hyogo Japan; 9 Department of Cardiology, Hokusetsu General Hospital, Takatsuki, Osaka Japan; 10 Department of Cardiology, Fukuoka Wajiro Hospital, Fukuoka, Japan; 11 Cardiovascular Center, Saiseikai Yokohama City Eastern Hospital, Yokohama, Kanagawa Japan

**Correction to: Cardiovascular Intervention and Therapeutics**

**https://doi.org/10.1007/s12928-026-01282-2**

In this article, Figs. 7 and 22 appeared incorrectly and have now been corrected in the original publication. For completeness and transparency, both the correct and incorrect versions are displayed below.

Incorrect Figs. 7 and 22:

**Fig. 7:**
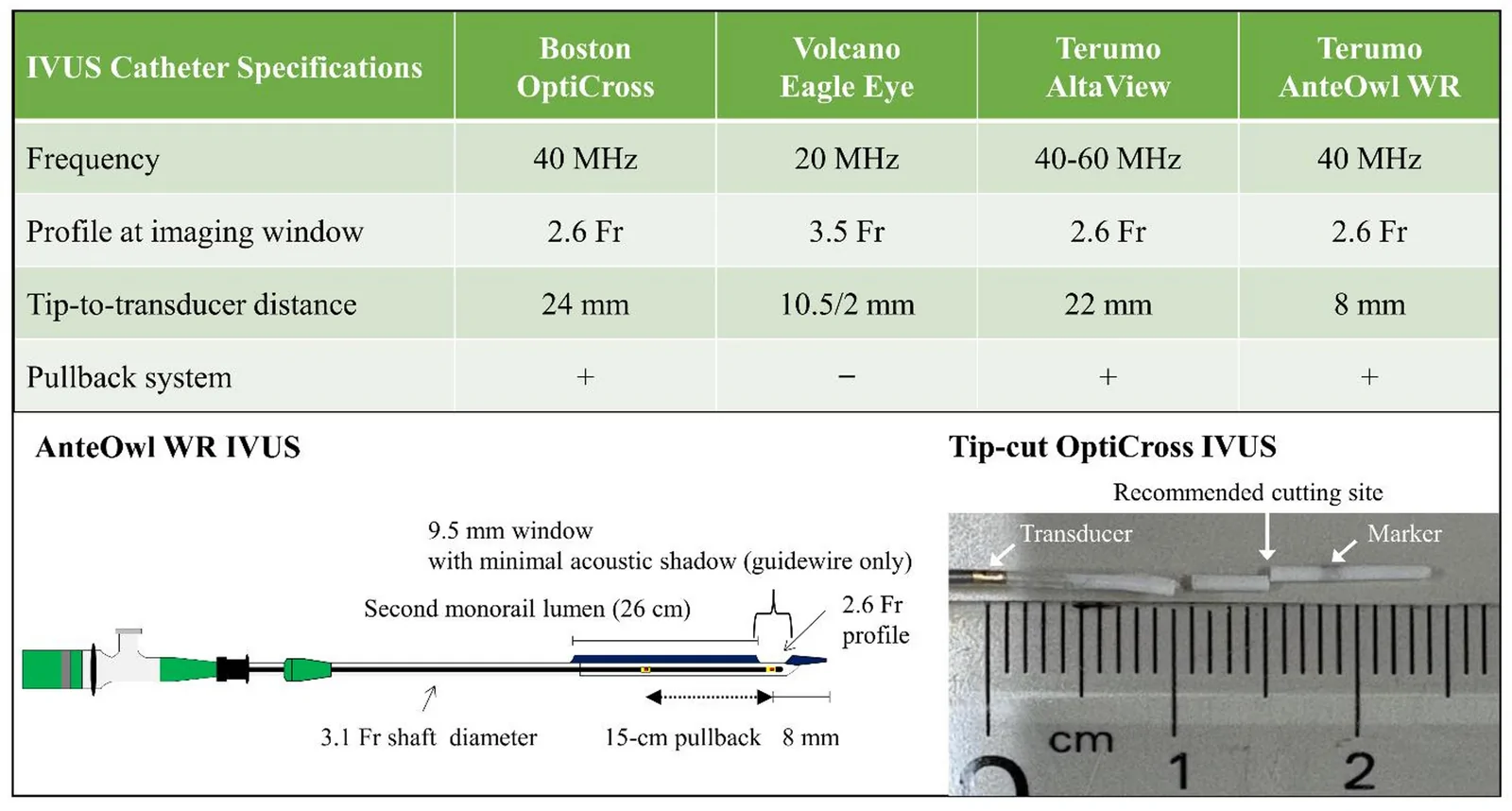
Comparison of Specifications: AnteOwl WR vs. Conventional IVUS Systems. Overview of key specifications for major IVUS systems and a detailed structural illustration of the AnteOwl WR IVUS. *IVUS* intravascular ultrasound

**Fig. 22:**
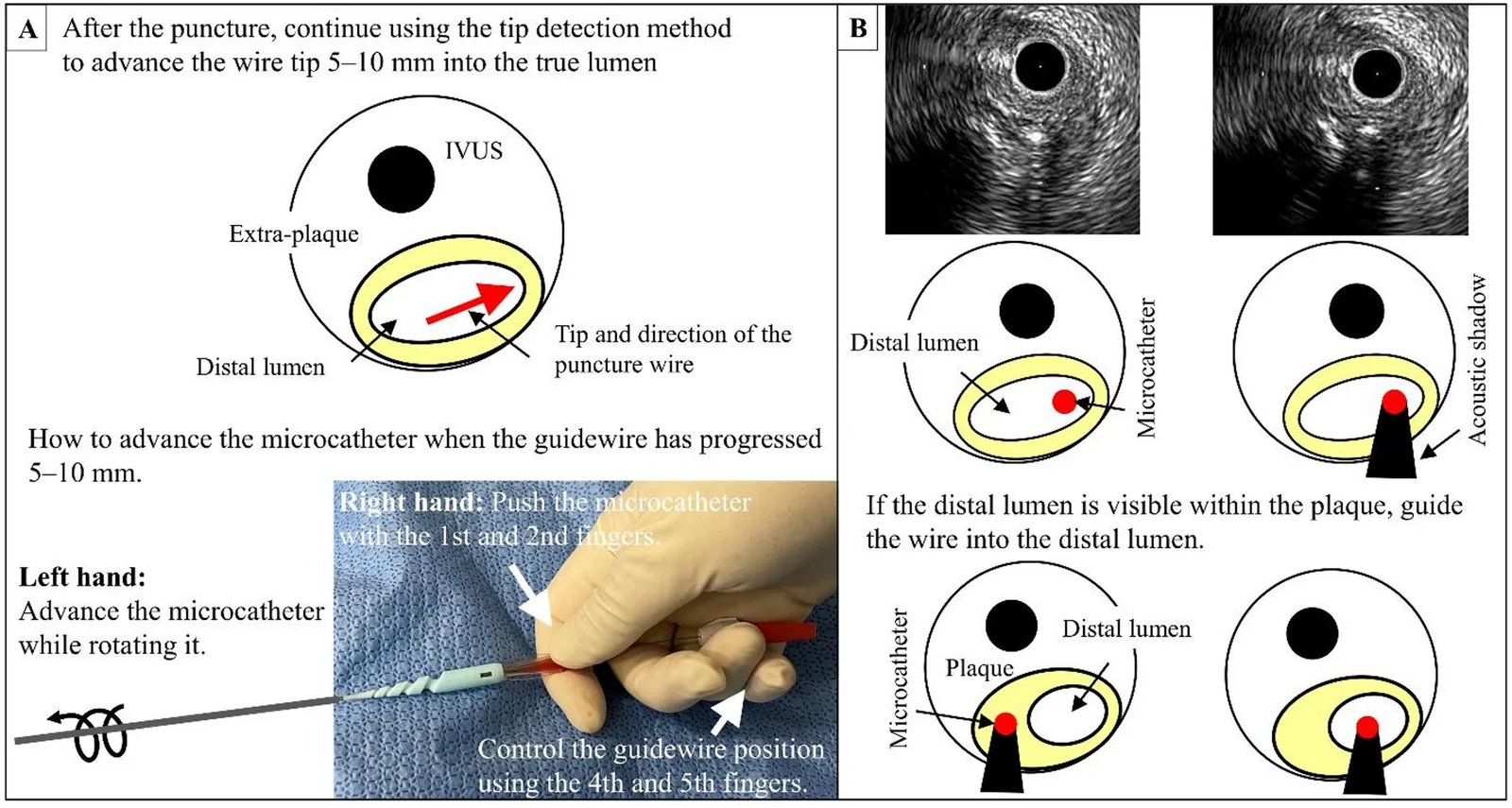
Advancing the microcatheter following puncture in Schematic illustration of the IVUS image and corresponding actual TD-ADR 　(**A**) Schematic illustrationof the IVUS image and a pho-IVUS images. *IVUS* intravascular ultrasound, *TD-ADR* tip detection-tograph showing hand maneuvers for advancing the microcatheter. (**B**) antegrade dissection re-entry

Correct Figs. 7 and 22:

**Fig. 7:**
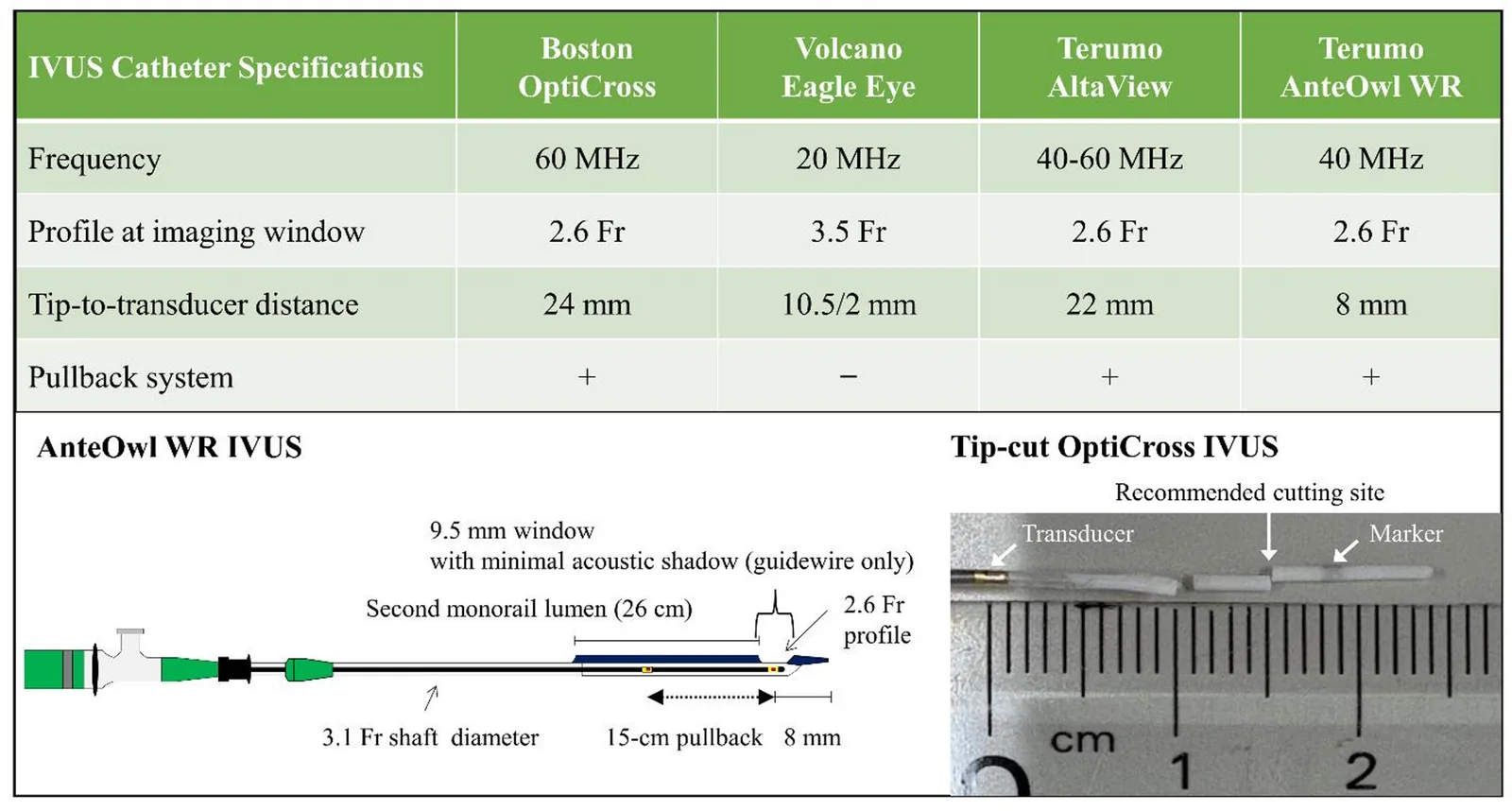
Comparison of Specifications: AnteOwl WR vs. Conventional IVUS Systems. Overview of key specifications for major IVUS systems and a detailed structural illustration of the AnteOwl WR IVUS. *IVUS* intravascular ultrasound

**Fig. 22:**
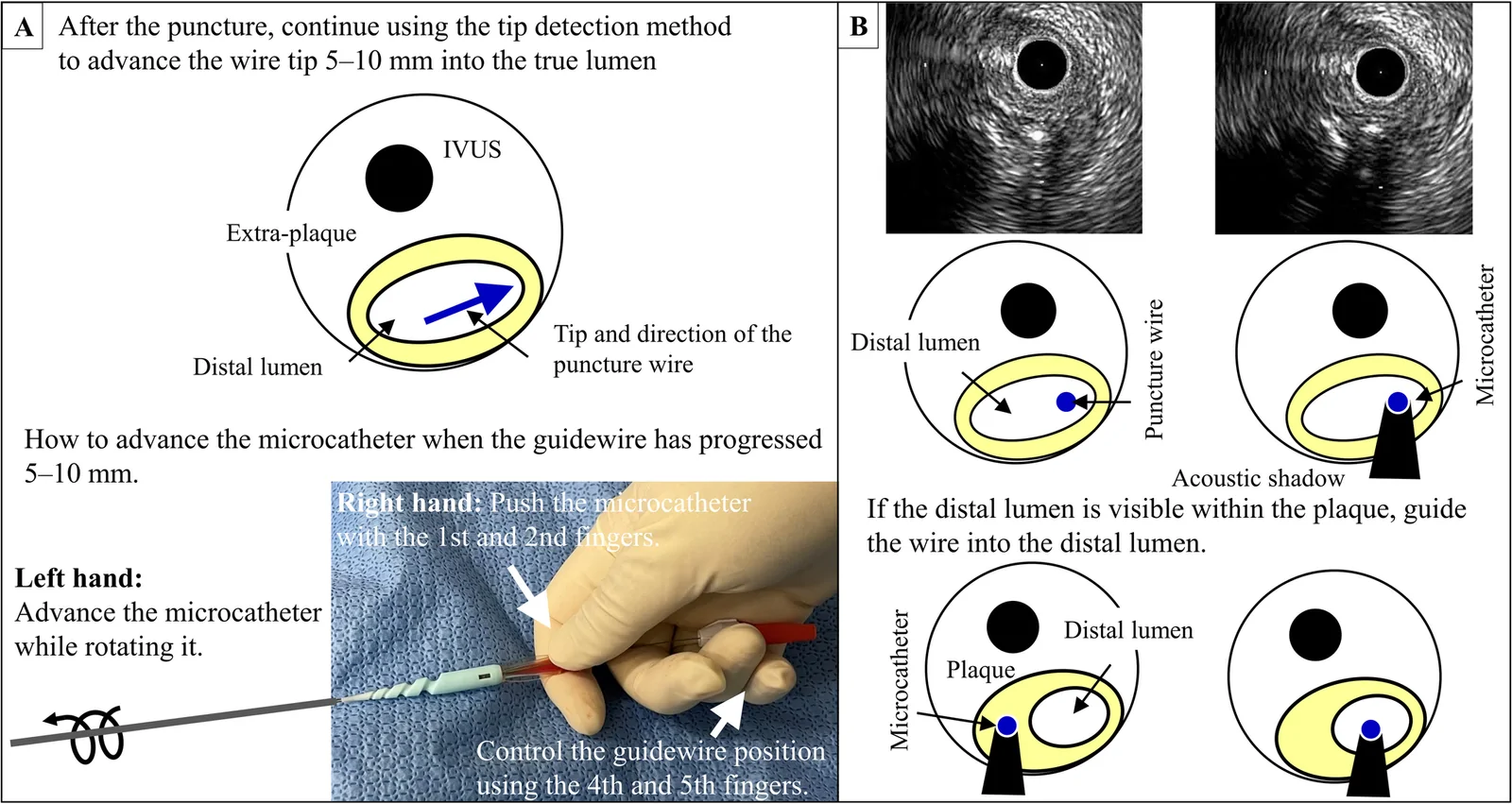
Advancing the microcatheter following puncture in Schematic illustration of the IVUS image and corresponding actual TD-ADR 　(**A**) Schematic illustrationof the IVUS image and a pho-IVUS images. *IVUS* intravascular ultrasound, *TD-ADR* tip detection-tograph showing hand maneuvers for advancing the microcatheter. (**B**) antegrade dissection re-entry

The original article has been corrected.

